# Effect of different postures and loads on joint motion and muscle activity in older adults during overhead retrieval

**DOI:** 10.3389/fphys.2023.1303577

**Published:** 2024-01-18

**Authors:** Chengmin Zhou, Xue Xu, Ting Huang, Jake Kaner

**Affiliations:** ^1^ College of Furnishings and Industrial Design, Nanjing Forestry University, Nanjing, Jiangsu, China; ^2^ Jiangsu Co-Innovation Center of Efficient Processing and Utilization of Forest Resources, Nanjing, China; ^3^ School of Art and Design, Nottingham Trent University, Nottingham, United Kingdom

**Keywords:** posture, comfort, muscle activity, range of motion, hanging cabinet

## Abstract

**Introduction:** Pain is a common health problem among older adults worldwide. Older adults tend to suffer from arm, lumbar, and back pain when using hanging cabinets.

**Methods:** This study used surface electromyography to record muscle activity and a motion capture system to record joint motion to research effects of different loads and retrieval postures on muscle activity and joint range of motion when older adults retrieve objects from a high place, to provide optimised feedback for the design of hanging cabinet furniture.

**Results:** We found that: 1) The activity of BB (Biceps brachii) on the side of the body interacting with the cabinet door was greater than that of UT (Upper trapezius) and BR (Brachial radius) when retrieving objects from a high place, the activity of UT on the side of the body interacting with a heavy object was greater than that of BB and BR. 2) The activity of UT decreases when the shoulder joint angle is greater than 90°, but the activity of BB increases as the angle increases. In contrast, increasing the object’s mass causes the maximum load on the shoulder joint. 3) Among the different postures for overhead retrieval, alternating between the right and left hand is preferable for the overhead retrieval task. 4) Age had the most significant effect on overhead retrieval, followed by height (of person), and load changes were significantly different only at the experiment’s left elbow joint and the L.BR. 5) Older adults took longer and exerted more effort to complete the task than younger adults, and static exercise in older adults may be more demanding on muscle activity in old age than powered exercise.

**Conclusion:** These results help to optimise the design of hanging cabinet furniture. Regarding the height of hanging cabinets, 180 cm or less is required for regular retrieval movements if the human height is less than 150 cm. Concerning the depth of the hanging cabinets, different heights chose different comfort distances, which translated into the depth of the hanging cabinets; the greater the height, the greater the depth of the hanging cabinets to use.

## 1 Introduction

In the 21st century, most countries have entered a rapidly aging society. Pain is a sign of an underlying pathological disorder, either an ailment or an injury. Pain includes acute pain and chronic pain, the former of which can develop into chronic pain if meeting certain factors. Chronic pain is one of the most common health problems among older adults worldwide (Treede et al., 2015; Larsson et al., 2017; Chang et al., 2022). Chronic pain typically lasts longer than 3 months, and prevalence ranges from 27% to 86% (Johannes et al., 2010; Docking et al., 2011; Wettstein and Tesarz, 2023). For example, MSDs (Musculoskeletal Disorders), to which older adults are prone, often cause chronic pain. Studies have found gender differences in pain responses, with women having higher mean subjective pain scores than men (Etherton et al., 2014) and a higher prevalence of pain in females and older adults (Tsang et al., 2008). Chronic pain affects the quality of life and daily mood of older adults, leading to problems with sleep, exercise, and socialisation and diminishing independence, which is more important for older adults (Zis et al., 2017; Goyal and Mohanty, 2022; Tsai et al., 2022). The most common pain locations in order of frequency include back and pelvis (34.1%), lower limb (30.7%), upper limb (13.4%), shoulder and neck (10.2%), chest and abdomen (4.6%), and head (3.9%) (Freburger et al., 2009; Johannes et al., 2010). Studies have shown that lower back pain (LBP), upper limb pain, and neck pain are associated with improper work postures and repetitive bending or lifting, exacerbating spinal strain (Bernard and Putz-Anderson, 1997; Hoogendoorn et al., 2000; Hoy et al., 2010). Also, during bending or lifting, poor posture or localised muscle responses are associated with external loads (Madinei and Ning, 2018). That is, body joints and muscle activities are affected by posture and movement (Granata and Wilson, 2001; Arjmand and Shirazi-Adl, 2005), the magnitude of the load (Elsayed et al., 2015; Beaucage-Gauvreau et al., 2021) and the associated muscle fatigue (Granata et al., 2004; Granata and Gottipati, 2008).

Among users of hanging cabinets, older adults face more serious physical problems (Lewanska et al., 2016). Tasks such as opening and closing doors and pulling out drawers in hanging cabinets, bookcases, and other furniture cause significant changes in body parts, such as bending, stretching, and lifting (Richter-Kluge et al., 2019; Norin et al., 2021; Simundic et al., 2023). Older adults with hanging cabinets experience health problems such as arm, lumbar, and back pain when lifting and lowering heavy objects from high places (Hoy et al., 2010; 2012; DePalma et al., 2012; Rohlmann et al., 2013). As a result, with age and deterioration of physical functions, older adults experience increased task difficulty when using cabinets, especially requiring large body movement changes during use (Bilodeau et al., 2001; Vandervoort, 2002; Avin and Law, 2011).

Hanging cabinet is the upper storage space that enhances the storage capacity of the space. It is typically used in kitchens and bathrooms of residence. However, with modern home renovation’s increased spatial planning capabilities, hanging cabinets can also be utilised in entryways, living rooms, bedrooms, balconies, and more. In addition, inpatient wards, convalescent homes and other houses with special purposes will also appear hanging cabinets. This phenomenon dramatically increases space utilisation and indicates that hanging cabinets are used frequently in people’s daily lives. However, when the frequency of use of hanging cabinets is too high and the time is too long, older adults will experience severe capacity loss or even dangerous accidents (Rohlmann et al., 2013; Norin et al., 2021). The cabinet design must incorporate humanised scientific data support and put forward solutions to practical problems in combination with older adults’ physiological and behavioral characteristics, reflecting the social care for them. These data generally include the physiological data, psychological emotions, objective conditions, and subjective preferences of older adults when using the hanging cabinet to optimize the design of the style, function, structure, interaction, and other dimensions of the hanging cabinet.

The difficulty places greater demands on the ergonomic design of hanging cabinets to reduce the burden of use and improve users’ quality of life (Bazazan et al., 2019). There are fewer studies on using hanging cabinets by older adults, focusing more on multi-joint movements for sit-to-stand (STS) during chair use and sedentary studies (Bonnet and Barela, 2021; Christensen et al., 2023). STS is an essential daily task for determining whether people can live independently, so it is used as a behaviour of use to study muscle activity and fatigue (Roldan-Jimenez et al., 2015) or in combination with furniture to simultaneously qualitatively and quantitatively study the behaviour of use with the design parameters (Goncalves and Arezes, 2012; De Carvalho and Callaghan, 2022). Bryanton and Bilodeau (2019) used surface electromyography (sEMG) to study changes in the lower limb muscle activity during STS in both older and younger adults, with muscle compensations occurring during knee extension movements, resulting in increased dependence on the ankle and hip. Shi and Zhang (2023) used 8-channel sEMG to study the behaviour of older adults when hanging objects and to analyse the range of comfortable operating heights appropriate for older adults in different height groups. This study provides appropriate design parameters for storage furniture for older adults of different heights, providing data and theoretical support for furniture design. In addition to daily life use behaviours, workers’ work behaviours are often linked to musculoskeletal disorders. The primary study focuses on muscle activity and joint angles to explore fatigue problems and risks, such as workers’ material handling, drivers’ long-distance driving, and factory assembly line production (Rasmussen et al., 2009; Yu and Wu, 2019; Fang et al., 2021; Firouzabadi et al., 2021; Skals et al., 2021). For example, Skals et al. (2021) studied the joint Angle and muscle activity of the trapezius lowering muscle and the erector spinalis longest muscle during manual material handling in supermarkets. Fang et al. (2021) assessed the fatigue of different muscles during manual material handling (MMH) and recommended appropriate postural bending angles. Firouzabadi et al. (2021) studied gender differences in MMH. Considering several studies, research on muscle fatigue and joint angle range of motion collect baseline data using sEMG, motion capture, and other devices (Bieleman et al., 2021; Kazemi et al., 2022). Monitoring human fatigue status relies solely on sEMG for fatigue recognition and classification, leading to unstable results and certain limitations (Cattarello et al., 2018). However, fatigue state monitoring has introduced sEMG and motion capture technology synchronously collecting data through the upper limb loaded opening and closing cabinet door experiment (Millard et al., 2019; Sawaguchi and Tanaka, 2019), where the two types of data could support the reliability of the results simultaneously.

This paper focuses on retrieving objects from high places for older adults with variables of retrieving postures and object loads. The methodology includes the following: EMG records human muscle activities, which can reflect the characteristics of muscles. Upper limb muscle fatigue can be studied from the time-domain and frequency-domain characteristics. The motion capture system records the relevant joint data of older adults to study the range of motion of joints. We decode the behavior from a three-dimensional level and analyse the joint movements in conjunction with the basic movements of the human body. Data such as human height, arm span dimensions, and joint range of motion can be used to design hanging cabinets. We hypothesised that: 1) Differences in postures will cause joint angle changes, thus affecting older adults’ muscle activity. 2) The mass of objects retrieved also affects the muscle activity of older adults. 3) Age, gender, and height affect joint angle and muscle activity.

## 2 Methods

### 2.1 Participants

43 healthy participants completed our experiment, divided into two groups: 23 older adults (12 females and 11 males, aged 57–78 years between 147 and 182 cm in height) and 20 younger adults (10 females and 10 males, aged 20–25 years between 160 and 183 cm in height). In order to ensure consistency between older adults and younger adults, we excluded three younger elderly participants (including two females and one male), and the selected 20 participants were all over 60 years old. Older participants were residents, retired or unretired professors who lived within the school and were recruited as volunteers. Younger participants were students. In order to ensure that our participants had adequate mobility and vision, an ability assessment test was given to older participants before the experiment. Adequate mobility refers to the self-care ability of daily life and essential motor ability. This experiment assessed the ability of daily living activities by walking flat and removing the shirt. Normal vision refers to the ability of the participant to perceive the presence of light and perceive the size and shape of objects. This experiment evaluated participants by subjectively asking and reading standard fonts in books and newspapers. Our participants had normal mobility and vision and were free of physical disabilities or medical conditions. Before starting the experiment, we measured the arm lengths of our participants: elderly (39–62 cm, M = 48.25 cm, SD = 5.22 cm) and young (42–67 cm, M = 49.65 cm, SD = 5.52 cm). The total duration of each participant was approximately 20 minutes, which included the experimental instructions, body metrics measurements, rest, experiment, and interview.

### 2.2 Instruments

#### 2.2.1 Surface EMG

**FIGURE 1:**
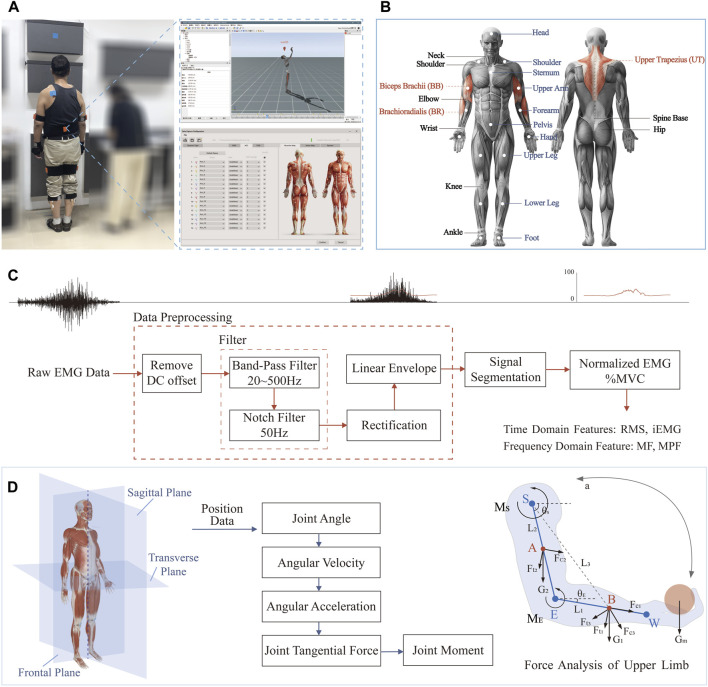
Experimental design and data processing flow. **(A)** The experimental shot and the software interface. **(B)** Locations of sEMG sensors. **(C)** Pre-processing of raw sEMG signals. **(D)** Motion capture data processing. Abbreviation: RMS (Root Mean Square), iEMG (integrate EMG), MF (Median Frequency), MPF (Mean Power Frequency), UT (Upper trapezius muscle), BR (Brachial radius muscle), BB (Biceps brachii muscle).

We recorded sEMG signals using a sEMG sensor with a sampling frequency of 2,000 Hz. First, we scrubbed the skin with alcohol cotton pads, and after the skin was dried, we placed the electrodes in the middle of the muscle at a distance of 2 cm along the direction of the muscle fibers. In the cabinet door’s opening and closing action, the upper limb’s main muscles that exert force are UT, BR, and BB. Therefore, we used six-channel sEMG signal acquisition to measure the muscles above the left and right sides, respectively, and Figure 1B shows the sensor positions.

#### 2.2.2 Motion capture

A real-time motion capture system, Xsens MVN Analyze, captured inertial motion at 17 nodes throughout the human body. The motion capture sensors were attached to the participant using a nylon attachment strap, a hair band, and a custom-made tight-fitting T-shirt with the sensor positions shown in Figure 1B. To initialize the sensors and establish a baseline estimate of the position and orientation of the body part, a calibration procedure had to be performed, which consisted of the participant standing in an upright position for a few seconds and then taking a few steps forward and backward to the starting position to complete the sensor calibration.

### 2.3 Experimental design

**FIGURE 2:**
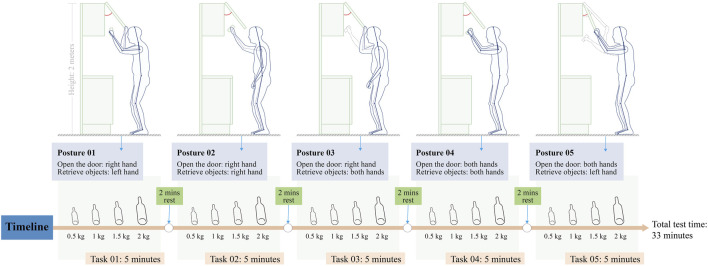
Task design of the experiment.

This experiment used retrieving posture and load configuration as two variables (from now on referred to as posture and load). The load consists of four classes: 0.5, 1, 1.5, and 2 kg. Each 0.5 kg is a test section. Considering the experimental environment and the size, shape, and mass of different objects, we chose plastic bottled beverages of different qualities as objects to retrieve (beverage bottles have the same shape, and the larger the size, the greater the mass). Postures consist of five types, defined as changes in the different combinations of the left and right hands (including the two stages of opening the cabinet door and retrieving the object), as shown in Figure 2 for details. The height of the hanging cabinet is set according to the typical height of the hanging cabinet and the human scale of older adults to ensure that the operation is generally consistent during the experiment to avoid older adults standing on their toes as far as possible. The height of the hanging cabinet ranges from the average visual range of older adults to the height of their hand function, corresponding to the scale of the hanging cabinet in this experiment, 1,800–2,000 mm. The top height of the cabinet is 2,000 mm, and the bottom height (off the ground) is 1,800 mm. The cabinet width is 500 mm. As for the selection of muscles for testing, since the changes in posture mainly come from the changes in arm movements and participants are all right-handed, the muscles mainly tested in this experiment are biceps brachii (BB), brachial radius (BR), and upper trapezius (UT), which play an essential functional role in the lifting movement. Participants were tested for no more than 30 minutes to prevent the physical and psychological discomfort experienced by older subjects.

The experimental area was 4 m × 9 m. Standard indoor lighting was maintained in the experimental area to provide a realistic indoor environment. The starting point and target location were marked on the floor for the participants to see when they looked down. The target location was directly under the hanging cabinet, which fixed at a height of 2 m. The participants walked from the starting point to the target location and to interact with the hanging cabinet according to the instructions (open the door–pick up an object–close the door). Before the hanging cabinet task, all participants signed an informed consent form and were informed of the procedure and details. The experimental task were divided into two categories according to the purpose of the experiment: the first category was the habitual posture experiment of the hanging cabinet, and the second category was the formal experimental task. For the habitual posture task, participants had to choose the most comfortable distance from the cabinet and stand to complete the movement. For the formal experimental task, participants must stand at the experimentally determined target point to complete the maneuver. The purpose of the habitual posture experiment is to understand the participants’ common postures when using a hanging cabinet in daily life and to analyse the optimal horizontal distance between participants of different heights when using the same hanging cabinet. In the habitual posture experiment, the participants were not instructed on postural styles, muscle use, or movement patterns.

### 2.4 Data analysis

#### 2.4.1 Electromyography

The EMG signal is a one-dimensional time series signal with a signal frequency of 0–500 Hz and a main signal frequency range of 20–150 Hz. EMG has a peak value between 0 and 6,000 *μ*V. When the muscle is completely relaxed, its baseline noise should be at 1–4 *μ*V, and the interfering frequency is mainly in the 0–60 Hz range. The density and height of EMG signals during muscle activity reflect to some extent the amplitude and force of muscle contraction (Okajima et al., 2023). Figure 1C shows the EMG signal processing flow. Integrated EMG (iEMG) expresses the total number of discharges from the motor units of the muscles involved in the activities in a given period. The greater the amplitude, the greater the fatigue. Root Mean Square (RMS) describes the average change in EMG over time. Muscle contribution determined the magnitude of the degree of muscle force generation. Median Frequency (MF) decrease as the duration of exercise increases. Mean Power Frequency (MPF) is the average value of frequency during the period.

#### 2.4.2 Motion capture

As shown in the left panel of Figure 1D, according to human anatomy, the plane of the human body includes frontal, sagittal, and transverse planes. Table 1 shows older adults’ three-dimensional action decoding (posture action decoding) in the behavioural stages of hanging cabinet use. The high retrieving action mainly occurs in the sagittal plane, so the sagittal plane is the primary research dimension. We were then establishing the ball-and-stick model of the human upper limb using the ball-and-stick structure with multi-rigid segments and hinge joints (Blache et al., 2015b) and defining the joint angles of the upper limb in the ball-and-stick model. Joint moments can directly show the loads borne at the joints and more accurately evaluate the joint comfort of the human body (Amarantini and Martin, 2004; Erdemir et al., 2007). The upper limb is divided into three parts, namely, the upper arm, forearm, and hand, and the details show in the right panel of Figure 1D.

**TABLE 1:** 3-D motion decoding. We decode the motion in 3-D and analyse the joint motion with the basic movements of the human body.

Motion plane	Task	Joint motion	Muscle	Diagram
Sagittal	Flexion/Extension	Shoulder Elbow Neck Dorsal Wrist Knee	BB BR UT	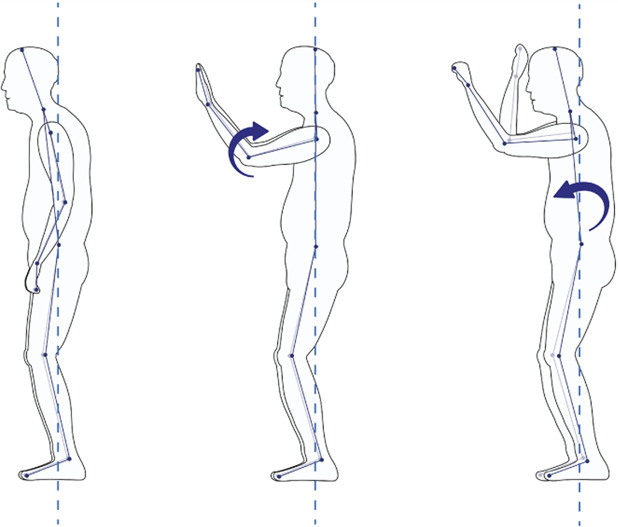
Frontal	Left/Right Abduction and Adduction	Forearm Scapula Shoulder	BR UT	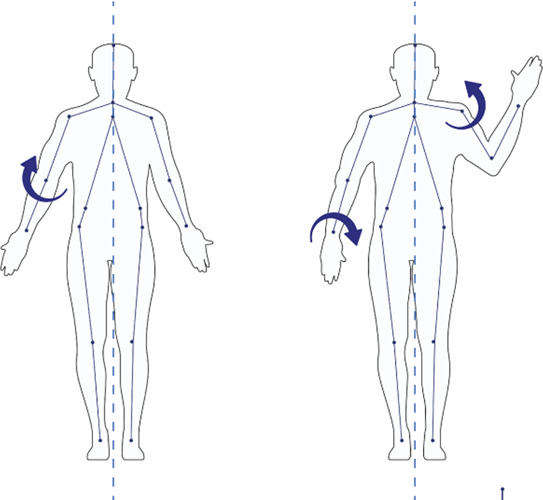
Transverse	Left/Right Internal Rotation and External Rotation	Scapula Shoulder	UT	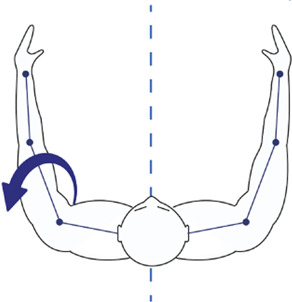

### 2.5 Statistics

For all statistical analyses, we used SPSS software to process the data. In the context of between-group comparisons, we generally preferred to use Levene’s test to examine variance alignment. Levene’s test *p* ≥ 0.05 means that the difference in variance between groups is not statistically significant, and the assumption of variance alignment is tenable. We used the Shapiro-Wilk test to check the normal distribution of the variables, and the assumption of the normal distribution of the data was valid if *p* ≥ 0.05. When the data passed the normal distribution test (*p* ≥ 0.05) and the variance chi-squared test (*p* ≥ 0.05), we performed a one-way analysis of variance (ANOVA) on the four quantitative EMG eigenvalues of each muscle under the different mass tasks to further test the effect of significance. Conversely, we use the Kruskal–Wallis test when the data shows a non-normal distribution (*p* < 0.05) or heterogeneous variance (*p* < 0.05). The same method was used for the kinematic and kinetic data statistical methods, including joint mobility and joint moments. The *χ* ^2^ test was used for count data, and the mean ± standard deviation was used to express measurement data, using the independent samples *t*-test between the two groups and the paired samples *t*-test within the group. The significance level in statistical analysis was defined as *p* < 0.05, indicating that the difference was statistically significant.

## 3 Results

### 3.1 Participant characteristics

#### 3.1.1 Basic information

Table 2 shows the descriptive statistics of the participants in this experiment. Based on the validity of the motion capture and sEMG data, 40 participants were screened and analysed, with older adults aged 60–78 years of age and younger adults aged 20–25. The gender distribution was even, with a 50% male-to-female ratio in both groups. In the study sample, the older group had a mean age (of 64.75 ± 4.85) years, height (of 164.88 ± 9.82) cm, and body mass (of 68.65 ± 5.26) kg, and the younger group had a mean age (22.55 ± 1.36) years, height (171.25 ± 7.56) cm and body mass (60.45 ± 7.65) kg. The height of the two groups (*t* = −2.301, *p* = 0.320), body mass (*t* = 3.948, *p* = 0.122), and body mass index (*t* = 8.393, *p* = 0.322) were compared without significant difference (*p* > 0.05). Age (*t* = 37.443, *p* = 0.014) was significantly different (*p* < 0.05) between the two groups.

**TABLE 2:** Comparison of general information between the two groups.

Groups	Num	Gender ratio	Age (years)	Height (cm)	Body Mass (kg)	BMI (kg/m^2^)
Older	20	1:1	64.75 ± 4.85	164.88 ± 9.82	68.65 ± 5.26	25.31 ± 1.50
Younger	20	1:1	22.55 ± 1.36	171.25 ± 7.56	60.45 ± 7.65	20.58 ± 2.02
T-value			37.443	−2.301	3.948	8.393
*p*-value			0.014	0.320	0.122	0.322

#### 3.1.2 Habitual posture clustering

Table 3 shows the cluster analysis results of the habitual postures of the 40 valid participants when using the hanging cabinet. Before beginning the formal experimental task, each participant needs to perform the opening and closing of the cabinet door and the retrieval task according to the habitual posture when using the hanging cabinet in everyday life. Finally, the habitual posture for retrieving objects from a hanging cabinet was analysed. The highest percentage of participants performed the task by alternating right and left hands (52.5%), followed by one hand (45%). In contrast, the percentage using both hands to complete the task was only 2.5%.

**TABLE 3:** Comparison of general information between the two groups.

Posture	Experimental posture	Frequency	Percentage	Effective percentage	Cumulative percentage
One-handed	Posture 02	18	45	45	45
Alternating hands	Posture 01	21	52.5	52.5	97.5
Both hands	Posture 03/04/05	1	2.5	2.5	100
Total		40	100	100	

#### 3.1.3 Body and cabinet distance

Table 4 shows the optimal horizontal distances for participants of different heights using a hanging cabinet obtained by iterative clustering. When people use hanging cabinets, they tend to choose the optimal distance for easy access to objects, and when the hanging cabinets are too high, and their height is not enough, they need to increase the height under their feet. The results show that if the participants’ height is less than 150 cm, a 2-m hanging cabinet is too high for them, and they need external aids to help them reach objects, such as adding mats. The optimal reaching distance was 10 cm when the height was between 154 and 157 cm, 20 cm when the height was between 158 and 167 cm, 30 cm when the height was between 168 and 177 cm, and 40 cm when the height was above 178 cm.

**TABLE 4:** The horizontal distance between a person and a hanging cabinet when using a hanging cabinet.

Height range (cm)	Horizontal distance (cm)	Adding mats
Below 150	—	yes
154–157	10	no
158–167	20	no
168–177	30	no
Above 178	40	no

### 3.2 Muscle activity

Table 5 shows the data on the EMG characteristics of the participants while performing the Posture 01 task. Of all the six muscles measured, the total variation in iEMG values (*μ*V) for the R.BR ranged from 5.0 to 6.5 *μ*V, reflecting that it produced the least muscle activity, with the L.BR being higher than the R.BR. Among the muscles on the left side, the L.UT had the highest activity, followed by the L.BB. Usually, the higher the amplitude of the iEMG, the greater the fatigue (RMS values also increase with fatigue). Therefore, it shows that the muscle activity evaluated according to the magnitude of the electromyographic time-domain eigenvalues when picking up a heavy object from a high place is BR < BB < UT, in order from smallest to largest, which is also the top-to-bottom direction of the arm muscles when the arm is held high.

**TABLE 5:** EMG time-domain and frequency-domain changes in different loads while performing posture 01.

	Muscle	Older group				Younger group			
		Mass .5 kg	Mass 1 kg	Mass 1.5 kg	Mass 2 kg	Mass .5 kg	Mass 1 kg	Mass 1.5 kg	Mass 2 kg
RMS	L.UT	13.47 (8.90)	12.99 (7.36)	12.82 (6.66)	13.52 (7.35)	14.02 (6.64)	13.96 (6.51)	14.94 (7.72)	15.54 (6.77)
	L.BR	7.28 (2.63)	8.48 (2.62)*	8.36 (2.82)*	9.10 (3.23)*	6.91 (2.39)	9.31 (3.70)*	10.38 (5.32)*	11.46 (5.50)*
	L.BB	12.63 (7.24)	11.79 (5.48)	12.29 (5.93)	12.71 (5.68)	5.42 (1.61)	5.58 (1.86)	5.97 (2.21)	6.48 (1.98)^*#*^
	R.UT	10.30 (3.95)	9.98 (3.77)	9.70 (3.78)	10.10 (3.84)	17.07 (11.15)^*#*^	17.02 (11.41)^*#*^	17.21 (11.39)^*#*^	17.15 (11.23)^*#*^
	R.BR	6.19 (2.88)	6.68 (3.52)	6.19 (3.66)	7.08 (3.81)	5.18 (1.49)	5.25 (1.76)	4.74 (0.75)	5.21 (1.06)
	R.BB	11.34 (3.18)	10.93 (3.67)	10.44 (3.43)	10.69 (2.99)	7.6 (4.13)^*#*^	7.52 (3.50)^*#*^	7.05 (2.89)^*#*^	6.85 (2.98)^*#*^
iEMG	L.UT	12.37 (7.81)	12.05 (6.82)	12.00(6.15)	12.57(6.30)	12.51 (6.28)	12.33 (5.88)	13.58 (7.35)	14.05 (6.39)
	L.BR	6.71(2.37)	7.59(2.43)	7.44(2.50)	7.91(2.65)*	6.48 (2.23)	8.11 (2.99)	8.90 (4.18)	9.74 (4.28)*
	L.BB	10.91(5.62)	10.51(4.60)	10.83(4.76)	11.26(4.75)	4.87 (1.53)	5.03 (1.65)	5.51 (2.09)	5.68 (1.76)^*#*^
	R.UT	9.70 (3.60)	9.46 (3.55)	9.28 (3.51)	9.61 (3.58)	16.15 (10.71)^*#*^	16.10 (10.85)^*#*^	16.42 (10.99)^*#*^	16.24 (10.59)^*#*^
	R.BR	5.65 (2.65)	5.99 (3.09)	6.08 (3.01)	6.24 (3.14)	4.58 (1.39)	4.63 (1.59)	4.30 (0.81)	4.52 (1.02)
	R.BB	10.25 (2.84)	9.94 (3.11)	9.60 (3.01)	9.71 (2.70)	6.71 (3.44)^*#*^	6.71 (3.20)^*#*^	6.25 (2.79)^*#*^	6.16 (2.78)^*#*^
MPF	L.UT	81.89 (10.18)	81.83 (9.46)	81.90 (10.13)	82.11 (10.04)	72.64 (7.46)^*#*^	73.57 (7.93)^*#*^	73.89 (8.69)^*#*^	74.22 (8.86)^*#*^
	L.BR	98.58 (14.30)	102.96 (14.00)	102.36 (12.77)	102.62 (10.27)	63.07 (8.04)	64.47 (8.33)	65.47 (8.39)	65.91 (8.28)
	L.BB	85.42 (17.07)	84.94 (15.85)	81.47 (14.88)	81.26 (16.44)	94.27 (13.52)	97.19 (12.59)^*#*^	106.11 (12.90)^*#*^	100.62 (10.77)
	R.UT	80.53 (9.97)	83.06 (10.48)	82.60 (9.98)	83.07 (10.52)	75.59 (9.67)	76.75 (9.00)	75.84 (8.48)	75.87 (8.81)
	R.BR	107.08 (22.09)	108.24 (22.02)	107.61 (20.37)	108.07 (20.27)	66.95 (9.60)	66.97 (8.02)	66.93 (7.82)^*#*^	66.96 (8.02)^*#*^
	R.BB	88.46 (12.08)	85.65 (13.25)	85.90 (14.29)	85.15 (14.74)	91.01 (13.72)^*#*^	90.33 (14.71)^*#*^	87.28 (15.09)^*#*^	87.80 (17.78)^*#*^
MF	L.UT	71.50 (10.05)	70.87 (9.47)	71.50 (8.71)	71.41 (8.90)	75.12 (14.73)^*#*^	77.59 (16.26)	88.42 (13.85)	81.95 (11.42)
	L.BR	81.60 (14.47)	86.66(15.34)	85.21(15.46)	87.79(13.11)	66.19(14.23)	68.74(17.28)	66.94(15.72)	69.42(16.83)
	L.BB	67.03 (14.20)	68.84 (14.02)	63.52 (14.65)	64.21 (15.74)	51.78 (12.10)^*#*^	52.63 (14.36)^*#*^	51.28 (13.28)^*#*^	53.28 (11.36)
	R.UT	70.30 (11.89)	73.67 (10.33)	73.05 (9.81)	73.56 (10.28)	71.62 (13.55)	71.48 (14.17)	67.29 (15.82)	68.53 (18.62)
	R.BR	89.11 (22.38)	89.62 (22.87)	90.07 (20.48)	90.46 (21.48)	75.60 (14.45)	71.42 (10.24)	75.56 (15.82)^*#*^	74.15 (13.12)^*#*^
	R.BB	72.60 (15.31)	69.92 (14.91)	70.26 (14.93)	69.45 (17.09)	58.40 (10.51)^*#*^	55.54 (8.50)^*#*^	55.71 (11.80)^*#*^	56.38 (9.51)^*#*^

“*“: indicates a significant difference (*p* < 0.05) compared to Mass .5 kg “*#*”: denotes a significant difference (*p* < 0.05) between groups.

ANOVA showed that, among all the muscles, only L.BR showed a significant difference (*p* < 0.05) in retrieving objects of different loads. In contrast, the differences in the results of the paired comparisons between the other groups were not statistically significant. The *t*-test showed that the comparison between the older and younger groups showed significant differences in specific muscles (*p* < 0.05). R.UT and R.BB showed significant differences in RMS, iEMG (*p* < 0.05). Significant differences were shown in the muscles on the non-force exerting side. During experimental Posture 01, the right arm always lifted the cabinet door until completing the task. During this process, the activity of R.UT in the older group was lower than that of, the younger group, while the activity of R.BB was higher than that of the younger group. As for the frequency domain eigenvalues, the MPF values of L.UT and R.BB showed significant differences (*p* < 0.05), and the MF of L.BB and R.BB showed significant differences (*p* < 0.05). Generally, the frequency domain eigenvalues decrease with moderate to high-intensity exercise, indicating local muscle fatigue with decreasing changes. From the MPF values, the L.UT of the older group was higher than that of the younger group. The R.BB was lower than that of the younger group, which was in line with the trend of the time-domain eigenvalues, and the older group was more susceptible to fatigue at the R.BB than the younger group. Regarding MF values, the L.BB of the older group was higher than that of, the younger group, and the R.BB was higher than that of the younger group. The older group was more susceptible to fatigue at R.BB than the younger group.

**FIGURE 3:**
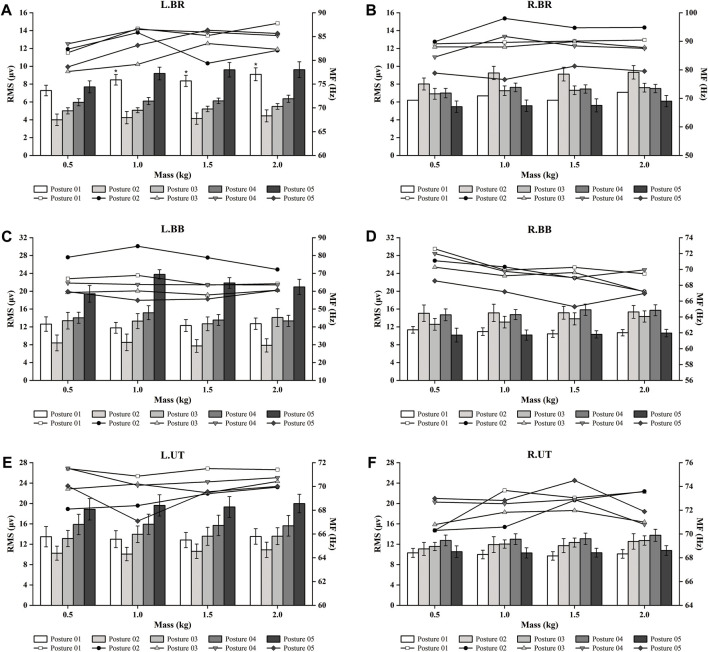
EMG time and frequency domain (RMS and MF) changes in different postures and loads. “*”: indicates a significant difference (*p* < 0.05) compared to Mass .5 kg. **(A)** RMS and MF changes of left BR. **(B)** RMS and MF changes of right BR. **(C)** RMS and MF changes of left BB. **(D)** RMS and MF changes of right BB. **(E)** RMS and MF changes of left UT. **(F)** RMS and MF changes of right UT.

Figure 3 shows the RMS values produced by the five posture tasks with different masses compared to the MPF values. For the L.BR activity (Figure 3A), the two tasks with the highest ranked RMS values were Posture 05 and Posture 01, with Posture 05 being higher than Posture 01. For the L.BB and L.UT activities (Figures 3C, E), the highest-ranked task was Posture 05. The highest-ranked task for the R.BR activity (Figure 3B) was Posture 02. For the R.BB activity (Figure 3D), there was no significant difference between the postures, and the two higher tasks were Posture 02 and Posture 04. The highest-ranked task for the R.UT activity (Figure 3F) was Posture 04. Compared to the RMS results for the BR, the BB and UT activities were higher in general, and there was a greater magnitude of variability between postures for the left muscles and a smaller magnitude of variability for the right muscles. However, as the object’s mass increased, the different muscle positions of the different postures did not produce significant changes, and only the RMS value of L.BR for Posture 01 was statistically significant (*p* < 0.05). The different object mass changes in the other postures did not show significant changes in all muscles. MF is the intermediate value of the discharge frequency during muscle contraction, which tends to decrease as the exercise time increases. The MF values of BR (range: 80–100 Hz) were higher than those of BB (range: 50–70 Hz) and UT (range: 60–70 Hz). The effect of different mass changes on MF values was not significant.

**FIGURE 4:**
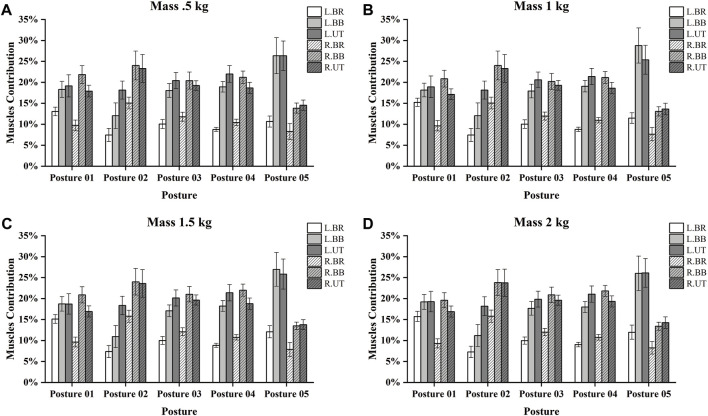
Ranking of muscle contribution in older adults with different postures and loads. **(A)** The rank when the load mass is 0.5 kg. **(B)** The rank when the load mass is 1 kg. **(C)** The rank when the load mass is 1.5 kg. **(D)** The rank when the load mass is 2 kg.

The muscle contribution ratio is a relative index, and the corresponding target muscles are selected to reflect the strength status of the movement. As shown in Figure 4, in Posture 01, R.BR has the most minor muscle contribution, and R.BB has the largest. In Posture 02, L.BR has the most minor muscle contribution, and R.BB has the largest. In Posture 03, the muscle contribution of L.BR was minimum, and that of L.UT was maximum when the loads were 0.5 and 1 kg, and that of R.BB was maximum when the loads were 1.5 and 2 kg. In Posture 04, the muscle contribution was the same as in Posture 03. In Posture 05, the muscle contribution of R.BR was the smallest, and that of L.BB and L.UT was the largest.

### 3.3 Joint range of motion

Range of Motion (ROM), is the maximum range of motion by a joint in a particular body position, also referred to as joint mobility. The maximum ranges (95% confidence intervals) of flexion/extension angles for the upper limbs, including the wrist, elbow, shoulder, neck and trunk, are listed in degrees in Table 6. The retrieving of objects from a high place in this experiment was within the normal values of joint mobility. In the wrist joint, the highest flexion angles requiring the left wrist joint were Posture 03 (6.5°), Posture 04 (6.45°), and Posture 05 (6.09°), and the highest flexion angles for the right wrist joint were Posture 05 (14.18°), Posture 03 (8.6.3°), Posture 01 (7.88°), and Posture 04 (7.76°). In the elbow joint, the highest flexion angle requiring the left elbow was Posture 05 (118.36°), followed by Posture 03 (110.33°) and Posture 04 (111.61°); the highest flexion angle for the right elbow was Posture 02 (108.53°). In the shoulder joints, the highest flexion angle required for the left shoulder joint was Posture 05 (122.51°), followed by Posture 04 (107.11°) and Posture 03 (99.51°); the highest flexion angle for the right shoulder joint was Posture 04 (107.06°). Of the neck joints, the one requiring the highest flexion angle was Posture 01 (27.95°). The highest flexion angle for trunk flexion was Posture 04 (6.01°).

**TABLE 6:** Joint range of motion of the upper limb in different posture in Sagittal dimension with 95% confidence interval.

Task	Wrist		Elbow		Shoulder		Neck	Trunk
	L	R	L	R	L	R		
**Posture 01**	4.83 (2.89–6.77)	7.88 (6.29–9.46)	102.25 (97.88–106.61)	101.54 (97.98–105.1)	91.73 (85.35–98.12)	104.01 (100.38–107.64)	27.95 (24.98–30.92)	5.66 (3.25–8.07)
Mass 0.5 kg	4.76 (0.97–8.56)	8.10 (4.59–11.62)	108.63 (102.72–114.54)	101.27 (94.40–108.14)	94.46 (82.51–106.40)	105.26 (97.53–112.98)	30.32 (23.83–36.81)	5.48 (0.32–10.65)
Mass 1.0 kg	6.32 (1.26–11.37)	7.99 (4.29–11.69)	109.44 (104.11–114.76)+	99.13 (92.01–106.25)	93.35 (82.07–104.63)	104.63 (96.86–112.39)	27.78 (21.62–33.34)	5.06 (−0.16–10.29)
Mass 1.5 kg	3.48 (−0.34–7.30)	9.37 (5.73–13.01)	99.30 (94.40–104.19)+	105.07 (95.62–113.52)	90.75 (74.15–107.34)	103.03 (95.10–110.96)	27.54 (20.90–34.19)	6.31 (0.98–11.63)
Mass 2.0 kg	4.76 (0.76–8.77)	6.04 (3.38–8.70)	91.62 (77.06–106.18)+	100.6 (92.52–108.85)	88.39 (73.32–103.45)	103.13 (94.86–111.39)	26.45 (19.70–33.20)	5.78 (0.44–11.11)
**Posture 02**	3.52 (1.93–5.11)	3.74 (1.95–5.54)	66.09 (52.06–80.11)	108.53 (105.5–111.57)	49.05 (37.18–60.92)	97.74 (93.34–102.14)	23.46 (20.5–26.42)	5.31 (3.2–7.43)
Mass 0.5 kg	4.58 (1.07–8.08)	3.39 (−0.21–6.98)	65.41 (33.42–97.40)	109.82 (103.65–116)	47.15 (21.49–72.80)	97.71 (88.49–106.93)	22.94 (16.52–29.36)	5.37 (0.97–9.77)
Mass 1.0 kg	3.66 (−0.91–8.23)	3.79 (−0.20–7.78)	63.72 (32.81–94.62)	109.42 (103.01–115.82)	46.23 (19.89–72.58)	97.41 (87.66–107.17)	22.28 (15.87–28.70)	5.38 (1.13–9.62)
Mass 1.5 kg	3.54 (0.48–6.59)	4.28 (0.07–8.50)	67.12 (36.98–97.27)	106.39 (99.41–113.37)	52.14 (26.18–78.10)	96.69 (86.69–106.69)	24.01 (17.67–30.36)	5.13 (0.12–10.13)
Mass 2.0 kg	2.32 (0.05–4.59)	3.51 (−0.36–7.37)	68.11 (38.63–97.58)	108.5 (101.73–115.27)	50.67 (24.99–76.36)	99.13 (89.7–108.57)	24.61(18.02–31.20)	5.37 (0.55–10.19)
**Posture 03**	6.5 (4.68–8.31)	8.63 (6.75–10.51)	110.33 (107.73–112.92)*	103.86 (100.73–106.99)	99.51 (95.48–103.54)*	102.79 (98.53–107.05)	22.08 (19.38–24.78)	5.52 (3.43–7.61)
Mass 0.5 kg	6.24 (3.33–9.14)	8.41 (4.88–11.95)	110.98 (104.45–117.52)	104.41 (98.04–110.79)	100.15 (90.35–109.94)	101.56 (92.11–111.02)	20.9 (15.14–26.66)	5.71 (1.63–9.79)
Mass 1.0 kg	6.83 (3.65–10)	7.85 (3.5–12.2)	111.98 (107–116.97)	102.64 (97.56–107.73)	99.57 (90.85–108.3)	101.54 (92.38–110.69)	21.81 (16.16–27.45)	5.71 (1.59–9.82)
Mass 1.5 kg	5.9 (1.81–9.99)	9.61 (5.25–13.98)	108.54 (103.26–113.83)	106.27 (97.84–114.7)	98.7 (90.49–106.91)	103.76 (95.08–112.44)	22.56 (16.56–28.56)	5.23 (0.47–9.99)
Mass 2.0 kg	7.03 (2–12.05)	8.66 (4.9–12.42)	109.8 (104.62–114.98)	102.12 (95.78–108.46)	99.62 (91.91–107.33)	104.29 (95.11–113.47)	23.06 (17.36–28.75)	5.42 (0.51–10.33)
**Posture 04**	6.45 (4.72–8.18)	7.76 (5.79–9.72)	111.61 (108.39–114.83)*	102.37 (99.92–104.82)	107.11 (102.07–112.14)*	107.06 (99.31–114.81)	22.39 (19.56–25.21)	6.01 (4.02–8.01)
Mass 0.5 kg	7.52 (3.97–11.06)	6.16 (2.93–9.4)	112.19 (105.72–118.67)	100.29 (96.11–104.47)	109.09 (98.34–119.85)	107.52 (97.37–117.67)	22.84(16.98–28.7)	6.22(2.16–10.28)
Mass 1.0 kg	6.34 (3.71–8.97)	6.59 (2.35–10.83)	111.21 (104.45–117.96)	103.4 (97.84–108.96)	107.56 (97.31–117.8)	104.68 (97.9–111.46)	20.58 (15.35–25.8)	6.08 (2.01–10.16)
Mass 1.5 kg	6.5 (2.37–10.63)	8.48 (4.11–12.85)	111.95 (104.62–119.27)	101.4 (96.62–106.18)	106.19 (95.36–117.02)	107.56 (97.32–117.81)	23.05 (16.19–29.91)	5.64 (1.16–10.13)
Mass 2.0 kg	5.45 (1.18–9.71)	9.8 (5.16–14.44)	111.1 (104.08–118.11)	104.41 (98.35–110.46)	105.59 (94.33–116.86)	107.06 (99.31–114.81)	23.08 (17–29.16)	6.11 (1.65–10.56)
**Posture 05**	6.09 (3.66–8.52)	14.18 (10.94–17.41)*	118.36 (112.21–124.51)*	103.38 (99.16–107.59)	122.51 (112.5–132.53)*	103.36 (97.04–109.69)	14.59 (11.86–17.33)**	4.58 (−0.17–9.34)
Mass 0.5 kg	6.43 (1.25–11.6)	15.23 (6.54–23.91)	119.89 (104.19–135.58)	107.36 (96.18–118.53)	122.16 (96.38–147.94)	103.38 (88.1–118.66)	14.16 (6.62–21.7)	5.6 (−5.04–16.24)
Mass 1.0 kg	7.2 (−1.29–15.68)	13.56 (4.06–23.06)	120.39 (106.17–134.6)	103.42 (89.81–117.02)	123.02 (97.29–148.76)	103.24 (86.98–119.5)	14.55 (9.03–20.08)	3.98 (−6.5–14.47)
Mass 1.5 kg	6.01 (−0.08–12.1)	13.14 (5.96–20.33)	117.64 (98.59–136.7)	101.57 (96.22–106.91)	123.31 (97.55–149.08)	103.69 (88.62–118.76)	14.57 (6.48–22.67)	3.68 (−8.97–16.33)
Mass 2.0 kg	4.73 (1.27–8.19)	14.78 (7.89–21.68)	115.54 (103.62–127.46)	101.17 (91.55–110.78)	121.57 (97.55–145.59)	103.15 (85.85–120.45)	15.09 (8.9–21.28)	5.07 (−8.86–18.99)

“*“: Compared with Posture 02, Postures 03, 04, and 05 are significantly different from it (*p* < 0.05). “**“: Compared with Posture 01, Posture 05 is significantly different from it (*p* < 0.05). “+”: compared with Mass .5 kg, other loads were significantly different from it (*p* < 0.05).

The ANOVA results showed that the independent variable of loads had a significant effect only on the joint mobility of the left elbow joint during the Posture 01 task. Furthermore, the independent variable of different postures significantly affected the left elbow and left shoulder joints. From the left elbow and left shoulder joints, there was a significant difference (*p* < 0.05) between Postures 03, 04, and 05 compared to Posture 02. From the right wrist joint, there was a significant difference between Posture 05 compared to Posture 02 (*p* < 0.05). From the neck joints, there was a significant difference between Posture 05 compared to Posture 01 (*p* < 0.05).

The maximum ranges (95% confidence intervals) of flexion/extension angles for the lower limb, including the ankle, knee, and hip, are presented in Table 7. There were no significant differences in either the ankle or knee joints. Among the hip joints, the highest flexion angles required for the left hip were Posture 03 (8.94°), Posture 04 (8.65°), and Posture 02 (7.56°); the highest flexion angles required for the right hip were Posture 03 (10.36°) and Posture 04 (9.49°). The ANOVA results showed that the different postures, the independent variable, significantly affected the hip joint. There was a significant difference between Postures 03 and 04 for the left hip joint compared to Posture 01 (*p* < 0.05).

**TABLE 7:** Joint range of motion of the lower limb in different posture states in Sagittal dimension with 95% confidence interval.

Task	Foot		Ankle		Knee		Hip	
	L	R	L	R	L	R	L	R
**Posture 01**	0.29 (0.11–0.46)	0.33 (0.24–0.42)	8.16 (7.55–8.76)	8.62 (7.8–9.43)	7.08 (5.78–8.39)	7.76 (6.45–9.08)	2.94 (1.37–4.51)	4.63 (2.83–6.42)
Mass 0.5 kg	0.18 (−0.11–0.47)*	0.37 (0.16–0.59)	7.91 (6.77–9.06)	8.62 (7.22–10.02)	6.22 (4.06–8.38)	6.92 (4.50–9.35)	2.98 (−0.55–6.52)	4.35 (0.65–8.04)
Mass 1.0 kg	0.27 (−0.11–0.65)	0.33 (0.12–0.54)	8.13 (6.96–9.30)	8.30 (6.81–9.78)	6.59 (4.25–8.93)	6.88 (4.54–9.22)	3.73 (0.14–7.32)	5.56 (1.46–9.67)
Mass 1.5 kg	0.29 (−0.06–0.65)	0.29 (0.15–0.44)	7.76 (6.75–8.77)	8.65 (7.12–10.18)	6.99 (4.25–9.73)	8.34 (5.55–11.12)	2.13 (−1.00–5.27)	4.24 (0.12–8.36)
Mass 2.0 kg	0.41 (−0.05–0.97)	0.33 (0.14–0.51)	8.83 (7.06–10.59)	8.89 (6.44–11.35)	8.54 (4.79–12.28)	8.90 (5.29–12.51)	2.91 (−0.49–6.30)	4.35 (0.65–8.05)
**Posture 02**	0.38 (0.29–0.46)	0.39 (0.18–0.59)	8.93 (8.2–9.67)	9.3 (8.19–10.41)	8.09 (6.39–9.78)	10.13 (8.02–12.25)	7.56 (5.9–9.21)	8.1 (6.37–9.82)
Mass 0.5 kg	0.32 (0.20–0.44)*	0.44 (0.01–0.87)	8.44 (7.06–9.82)	9.91 (7.25–12.57)	8.10 (4.33–11.86)	10.07 (5.78–14.36)	8.61 (5.04–12.17)	9.13 (5.21–13.04)
Mass 1.0 kg	0.33 (0.20–0.47)	0.44 (−0.06–0.94)	8.88 (7.38–10.38)	9.45 (6.88–12.02)	8.00 (3.88–12.12)	10.61 (5.26–15.95)	7.64 (3.60–11.67)	8.49 (4.02–12.96)
Mass 1.5 kg	0.38 (0.19–0.58)	0.21 (−0.22–0.64)	9.08 (7.41–10.75)	9.15 (6.81–11.48)	8.04 (4.44–11.63)	10.59 (5.90–15.28)	7.33 (3.28–11.38)	7.65 (3.88–11.42)
Mass 2.0 kg	0.47 (0.23–0.72)	0.45 (0.00–0.90)	9.33 (7.52–11.13)	8.69 (6.68–10.70)	8.22 (4.94–11.50)	9.27 (5.28–13.25)	6.66 (4.13–9.19	7.12 (4.57–9.67)
**Posture 03**	0.68 (0.46–0.9)	0.66 (0.5–0.82)	8.96 (8.13–9.79)	8.82 (7.84–9.8)	8.38 (6.63–10.13)	9.34 (7.48–11.21)	8.94 (6.9–10.98)+	10.36 (8.27–12.45)+
Mass 0.5 kg	0.5 (0.25–0.74)	0.65 (0.34–0.96)	9.3 (7.25–11.35)	8.31 (6.28–10.35)	9.01 (4.6–13.41)	9.43 (5.11–13.75)	9.66 (4.84–14.49)	11.41 (6.22–16.6)
Mass 1.0 kg	0.7 (0.08–1.31)	0.68 (0.34–1.02)	8.55 (6.89–10.2)	8.59 (6.47–10.71)	7.44 (3.85–11.03)	9.14 (5.23–13.05)	9.05 (4.59–13.51)	10.26 (5.6–14.91)
Mass 1.5 kg	0.67 (0.22–1.11)	0.54 (0.24–0.83)	8.87 (7.32–10.41)	8.42 (6.46–10.38)	8.47 (5.11–11.83)	9.11 (5.43–12.79)	7.88 (3.81–11.95)	9.02 (5.17–12.86)
Mass 2.0 kg	0.87 (0.42–1.33)	0.77 (0.35–1.18)	9.13 (7.32–10.94)	9.96 (7.78–12.13)	8.61 (5.1–12.13)	9.69 (5.65–13.73)	9.18 (5.12–13.23)	10.75 (6.74–14.77)
**Posture 04**	0.67 (0.51–0.84)	0.51 (0.36–0.66)	8.76 (8.04–9.49)	8.91 (8.02–9.81)	8.18 (6.48–9.88)	8.83 (7.08–10.59)	8.65 (6.47–10.83)+	9.49 (7.19–11.79)+
Mass 0.5 kg	0.83 (0.38–1.29)	0.64 (0.28–1)	8.86 (7.15–10.57)	9.03 (6.88–11.18)	8.75 (4.37–13.14)	9.1 (4.4–13.8)	9.9 (4.94–14.85)	10.69 (5.2–16.19)
Mass 1.0 kg	0.62 (0.28–0.96)	0.49 (0.24–0.73)	8.56 (7.05–10.07)	8.47 (7.02–9.92)	7.57 (4.07–11.07)	6.86 (4.75–8.97)	9.1 (4.78–13.43)	8.98 (3.96–14.01)
Mass 1.5 kg	0.47 (0.25–0.69)	0.37 (0.23–0.5)	8.68 (7.23–10.13)	8.92 (7.04–10.79)	8.04 (4.72–11.36)	7.98 (5.39–10.58)	6.42 (2.03–10.81)	7.34 (3.58–11.11)
Mass 2.0 kg	0.78 (0.44–1.11)	0.55 (0.11–1)	8.96 (7.4–10.52)	9.24 (7.12–11.35)	8.35 (5.1–11.59)	11.39 (6.86–15.93)	9.18 (4.35–14.01)	10.94 (5.89–15.99)
**Posture 05**	0.42 (0.05–0.8)	0.69 (0.12–1.25)	7.92 (6.26–9.57)	9.11 (7.29–10.93)	8.43 (4.12–12.74)	8.78 (4.81–12.75)	5.64 (1.59–9.69)	7.07 (3.14–11)
Mass 0.5 kg	0.55 (−0.9–2.01)	0.52 (−0.19–1.24)	6.88 (4.46–9.31)	8.12 (5.43–10.81)	5.57 (0.03–11.1)	6.38 (2.33–10.43)	2.97 (−3.23–9.16)	5.95 (−1.71–13.61)
Mass 1.0 kg	0.17 (0–0.33)	0.21 (0.05–0.37)	7.91 (3.44–12.38)	8.6 (5.39–11.81)	7.47 (−2.39–17.33)	7.52 (0.51–14.54)	6.87 (−3.38–17.13)	8.18 (−1.68–18.05)
Mass 1.5 kg	0.25 (0.09–0.41)	1.14 (−1.02–3.31)	7.73 (3.2–12.25)	9.85 (4.11–15.6)	7.93 (−1.56–17.42)	10.09 (−0.32–20.5)	4.51 (−4.98–14)	6.86 (−4.45–18.16)
Mass 2.0 kg	0.72 (−0.41–1.84)	0.87 (−0.71–2.46)	9.15 (4.51–13.78)	9.87 (4.24–15.49)	12.75 (−2.4–27.91)	11.11 (−3.55–25.77)	8.23 (−4.79–21.25)	7.29 (−3.11–17.69)

“*“: compared to posture 04, postures 01 and 02 are significantly different. “+”: compared to posture 01, postures 03 and 04 are significantly different from it.

### 3.4 Joint moment

**FIGURE 5:**
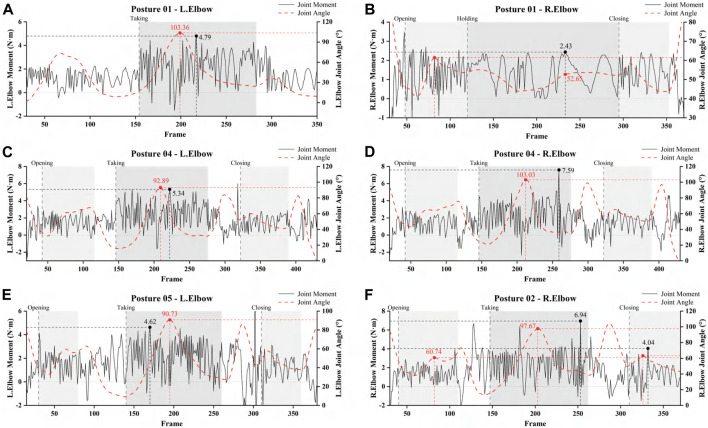
Elbow moments vs. elbow angle for different posture tasks. **(A)** The moment and angle of left elbow of Posture 01. **(B)** The moment and angle of right elbow of Posture 01. **(C)** The moment and angle of left elbow of Posture 04. **(D)** The moment and angle of right elbow of Posture 04. **(E)** The moment and angle of left elbow of Posture 05. **(F)** The moment and angle of right elbow of Posture 05. Note: Posture 03, which uses both hands to pick up objects, is highly similar to Posture 04, so Posture 04 was chosen as a representative.

The elbow joint dynamics of the upper limb movement when retrieving an object from a high place was investigated based on moment balance. Posture 01 is to implement the task of opening and closing the cabinet door with the right hand and retrieving objects with the left hand. Figures 5A, B show that the elbow joint angle of the upper limb changed a lot during the process of retrieving the object (up to 103.36°), and the joint moment is the maximum (4.79 N⋅ m). The maximum moment occurs at the elbow joint pinch angle of about 60°. During the movement transition, the moment was maximum in retrieving the object. While the right elbow joint is divided into three task states: opening the door–holding the cabinet door–closing the door, the maximum joint moment is generated during the opening phase, and the change in the joint angle was flat when keeping stationary against the cabinet door, with a maximum joint moment of 2.43 N⋅ m. Posture 04 task is to open the door and retrieve the object with both hands simultaneously. From Figures 5C, D, we can see that the changing trend of the elbow joints on both sides is the same, but the angle change of the right elbow joint is larger than that of the left elbow joint. Compared with Posture 01, the moment of Posture 04 is larger than that of Posture 01, and the two-handed task is more difficult than the one-handed task. Posture 05 task is to open the door with both hands and retrieve the object with the left hand, and the maximum moment is 4.62 N⋅ m, as can be seen from Figure 5E, which is similar to that of Posture 01 compared with one-handed retrieving the object. Posture 02 task is done with only one hand (right hand), and the maximum moment in the retrieving phase was 6.94 N⋅ m, and the maximum joint angle was 97.67°. This is close to the peak variation of Posture 04 (two-handed completion of the task). Taken together, the full two-handed completion of the task and the full one-handed completion resulted in greater moments and greater joint angle changes than the alternating right- and left-handed completion.

## 4 Discussion

This study investigated older adults’ muscle activity and joint motion during overhead retrieving with different postures and loads using a hanging cabinet furniture as the experimental vehicle. The main objective of this study was to find out whether different hand loads and retrieving postures during overhead retrieving would affect muscle activity and associated joint stability during load bearing to provide optimisation suggestions and control feedback for the design of hanging cabinet. The BR, BB, and UT muscles (a total of six points on the left and right sides) were chosen as the focus of the study. The joints of the body’s upper and lower limbs were examined, focusing on the upper limbs (including the wrist, elbow, shoulder, neck, and trunk). It was found that Postures 03 and 04 were greater than those of Posture 01 regarding trapezius muscle activity and range of joint angle variation. Posture 02 exacerbated right-hand fatigue, which was greater in muscle activity than in Posture 01. Therefore, alternating between the right and left hand was preferred for picking up objects from a high place. After excluding the differences in posture, the order of muscle activity from smallest to largest on the left side was BR, BB, and UT. In contrast, on the right side were BR, UT, and BB, which were related to the movement of the joints where the muscles were located. In conjunction with the kinetic study, the muscle activity of the trapezius muscle showed a decreasing trend, and the biceps muscle showed an increasing trend when the shoulder joint angle was greater than 90°. In the experiment, all participants were right-handed, so the experiment was set up so that they tended to open the cabinet door with their right hand and pick up the objects with their left hand, which caused a difference in muscle activity between the right and left sides. Together with other influencing factors, age had the greatest effect on picking up objects from a high place, followed by height, which was influenced by older adults’ comfort parameters when using a hanging cabinet (horizontal distance from the cabinet, need for external assistance to increase height). At the same time, differences such as mass and gender were insignificant in the low-load task. Finally, the design optimisation of hanging cabinet furniture is concluded based on behavioural studies of older adults. Table 8 shows the analysis and comparison between this paper and other literature.

**TABLE 8:** Key descriptors and findings extracted from reviewed scientific literature investigating upper limb movements.

References	Participants (M:F)	Mean age	Experimental method	Task	Variables	Muscle/Joints	Performance metric	Findings
Nielsen et al. (1998)	11 (male)	25 (21–29)	EMG Heart rate	Repetitive lifting of transport boxes (10 kg)	Lifting height-categories low:36–54 cm medium:72–126 cm high:144–163 cm Lifting frequencies 6/12/18 lifts per minute	MES MTR	RMS Mean muscular load (% MVE) Heart rate load	The maximum load on the shoulders was largest when lifting from the high lifting height (24.2% MVE at 18 lifts per minute)
Au and Keir (2007)	16 (1:1)	25.1 ± 1.8	EMG Force sensor Grip dynamometer Electrogoniometer	Hand and shoulder exertions	Grip conditions no grip 30% gripwith lowprecisionwith high recision maximal gripShoulder loading conditions maintaining posture 40%force-controlledmomentposture-controlledmomentMental loading stroop test/no stroop test	TR AD MD PD FCR FDS ECU EDC	Relative grip forces Shoulder moments Muscle activity mean muscular load (% MVE)	Simultaneous grip and shoulder exertions with additional demands of mental processing function to interactively increase muscle activity in the shoulder and forearm
Brookham et al. (2010)	10 (1:1)	23.2 ± 1.2	EMG Hand dynamometer Force transducer	Light hand tool exertions	Shoulder flexion 0°, 60°, 70°, 80°, 90° Shoulder rotation 0°, −45°, +45°	STR, MTR ITR, INF PM, LD AD, MD, PD	Muscle activities	TR decrease activation during 90° flexion, but deltoid activity continue to increase as flexion angle increased
Antony and Keir (2010)	16 (1:1)	25.3 ± 1.4	EMG	Isometric and dynamic shoulder exertions	Shoulder angles 30°/60°/90°/120° Loads no load/0.5 kg grip grip force of 30% of max Planes 0°/45°/90° Movement Speed slow/fast	AD MD PD PM INF LD BB TR	Mean muscle activity (% MVE)	Gripping increased BB activity by 6% MVE
Keir and Brown (2012)	20 (1:1)	25.8 ± 5.1	EMG	Cyclic bimanual pushing tasks	Push loads 1/2/4 kg Frequencies 4/8/16 min Grip conditions no required grip 30% of maximum grip force	PD, AD BB, TB ED, ECR FDS, FCR	Grip force Muscle activity mean muscular load (% MVE) Ratings of perceived exertion	Mean muscle activity increased with load, frequency and the addition of gripping. In the forearm, muscle activities were generally low
Martinez et al. (2020)	40 (1:1)	Women 21.4 ± 1.9 Men 24.9 ± 3.2	Force sensor Motion capture EMG	Moving an box between two shelves	Phases pulling (1%–20%) lifting (21%–60%) dropping (61%–100%) Mass 6/12 kg	BB, CORB AD, MD, PD INF, LVS PM, RM, SA TB, TR, LD	Muscle forces Muscle activations	Women generated higher muscle forces and activations when working above shoulder level
This work	40 (1:1)	Older 64.75 ± 4.85 Younger 22.55 ± 1.36	EMG Motion capture	Retrieving objects from high place	Postures five postures Mass 0.5/1/1.5/2 kg	Muscle UT, BR, BBJoints wrist, elbow shoulder neck, trunk hip, knee foot, ankle	Joint angle Joint moment Muscle activity	Posture 01 is the least effort. UT activity decreases when shoulder flexion is greater than 90° but BB activity increases as the angle increases. Increasing mass causes the maximum load on the shoulder joint. Mass and gender are insignificant in low-load task

“**“: indicates significant correlation at the 0.01 level (two-tailed).

### 4.1 Postures and loads

Regardless of the postural differences, the muscle activity order on the left side was BR, BB, UT, and on the right side, it was BR, UT, BB. Combined with the joint angles, the maximum angle range for opening the cabinet door was between 97.74° and 107.06°. In previous studies, when the elbow joint angle was 90°, the muscle activation level of the upper trapezius muscle stabilised when the shoulder joint angle reached between 60° and 90°, gradually increased between 30° and 60° and showed a decreasing trend when it was greater than 90°. However, the biceps muscle has been showing an increasing trend with the angle increase (Brookham et al., 2010). One of the reasons for this is that when the shoulder joint moves at a larger angle, it drives other parts of the body, such as the thorax, to compensate, which also explains the difference in the ordering of muscle activity between the right and left sides, as the whole arm (right) was raised upwards during the opening of the cupboard door, with a greater range of motion than the other arm (all participants were right-handed, and therefore the right hand was involved in the “opening of the cupboard door” step in all five postural tasks). The situation is different for the hand that holds heavy objects, as Nielsen et al. (1998) have found that the maximum load occurs at the shoulder when lifting objects from a height. The grasping motion of the hand caused an increase in EMG at the shoulder and a significant increase in arm muscle activity (Au and Keir, 2007), the same as the results of the present study. It suggests that complex tasks lead to a redistribution of muscle forces in a particular area, with multiple muscles synergistically participating in the movement (Umehara et al., 2021; Saito et al., 2022). In the arm’s flexion, the BB is the active muscle, and the BR is the secondary active muscle. However, muscle activity in the forearm is usually low (Keir and Brown, 2012). In the upward arm movement, the trapezius muscle acts as the active muscle (Roman-Liu and Tokarski, 2005). Thus, increased loading of the hand results in increased trapezius and biceps activity. Antony and Keir (2010) suggested that increased biceps activity during grasping objects may be the initial cause of altered muscle activity in the shoulder.

In addition, studies have shown that the average shoulder moment during unilateral and bilateral lifting is twice that of conventional extension (Joseph et al., 2014; Castelein et al., 2017). In this experiment, Posture 03 and Posture 04, which were two-handed for heavy lifting, had greater left and right trapezius activity than Posture 01, and it was seen that full unilateral and full bimanual were harder to accomplish the task than alternating left and right hands. Supplemented by the joint range of motion data (Tables 6, 7), the angular range at the hip joint was significantly greater for Posture 03 and Posture 04 compared to Posture 01. Figure 5 shows that the elbow joint moment was greatest in picking up the object during the movement transition. In contrast, in the movement interacting with the door of the cabinet (opening the door-pressing against the door-closing the door), the opening of the door phase produced the greatest moment. Also, the full two-handed completion of the task and the full one-handed completion resulted in greater moments and greater changes in joint angles than the alternating right- and left-handed completion. Therefore, alternating right and left hands for retrieving objects from a high place is more desirable, and the study results can help optimise the design and force feedback control of furniture such as hanging cabinets.

Martinez et al. (2020) found that musculoskeletal loading was higher in women than men during the lifting task. However, they also mentioned that this difference could be ignored if the mass was controlled in a way that was insufficient to create a strength difference (Bouffard et al., 2019). This experiment did not require direct involvement of the lower limb muscles, and the mass change was insufficient to cause excessive stress. However, Kim et al. (2003) have suggested that lower limb involvement can reduce the load-bearing stress on the upper limbs. As the object’s mass increased, only the RMS values of the L.BB muscle and the range of motion of the left elbow joint of the L.BR in posture 01 were statistically significantly different (*p* < 0.05). However, in the muscles that can be considered the main contributors to retrieving objects from high places (BB and UT), the muscle activity with increasing mass of the object did not show an increasing trend from low to high. Furthermore, the three left muscles in the youth group tapered in a low to high order in RMS and iEMG eigenvalues (e.g., Table 5), whether this means that the age difference between older and young adults in the overhead retrieving activity contributes to this, i.e., the reduced muscle sensitivity to the heavier objects resulted in, the older group not showing a more pronounced degree of muscle activation while holding the fourth set of heavy objects (heavier objects) (Deschenes, 2004). Arjunan and Kumar (2013) found that the older the age, the lower the sEMG complexity and the increased variability in muscle contractility. Older adults take longer to complete tasks than younger adults, exerting higher levels of effort as they age (Hortobagyi et al., 2003; Tikkanen et al., 2016). Muscle fatigue increases the harder and longer the human muscle exerts itself (Marras et al., 2006). Skeletal muscle is divided into slow muscle and fast muscle by the different ratios of the composition of fast and slow muscle fibers, and the anti-fatigue ability of slow muscle fibers is stronger than that of fast muscle fibers. However, its contraction force and speed are smaller than fast muscle fibers (Kern et al., 2001). Referring to the changing pattern of MF values (Figure 3), older adults produced the least fatigue at the BR during retrieving from a high place, and the main fatigued muscles were the BB and UT (UT > BB). Regarding muscle contribution, BB and UT dominated the overhead object retrieving maneuver (e.g., Figure 4), with specific left- and right-side differences depending on the posture. For example, in Posture 02, throughout the right hand, the right-side muscles were significantly larger than the left-side muscles. Table 5 shows that in Posture 01 in the muscles on the non-force exerting side, the older group showed significant differences from the younger group (R.UT and R.BB), with the older group being more prone to fatigue at R.BB than the younger group. Kak et al. (2019) found that the level of muscle activation was significantly higher under dynamic loading than under static loading but that this difference decreased with increasing load. Therefore, static exercise may be more depleting of muscle activity in the older age group than dynamic exercise on the left side of the body. In contrast, the younger age group is more dominant in dynamic exercise.

In conclusion, the analysis of different postures and loads leads to the following points: First, during retrieving objects at height, the activity of BB was greater than that of UT and BR on the side of the body interacting with the cabinet door, and the activity of UT was greater than that of BB and BR on the side of the body interacting with the heavy object. The difference in the different sides is that the activity of UT decreases for shoulder movements greater than 90°, but the activity of BB grows with the angle. At the same time, an increase in the mass induces a maximal loading at the shoulder joint. Second, among the different postures of retrieving objects from a high place, completing the task with both hands throughout and completing the task with one hand throughout was associated with greater muscle activity and moments, and greater changes in joint angles than alternating between the left and right hands, so alternating between the left and right hands to complete the task of retrieving objects from a high place was a more desirable choice. The heavy object retrieving and cabinet door opening phases were the most consumed during the maneuver. Three, gender differences were insignificant in the low-load task (loads in this experiment ranged from 0.5 to 2 kg) and were almost negligible. Fourth, mass changes were only significantly different at the left elbow joint and L.BR in the experiment because BR was the active muscle of movement in the hand when retrieving objects. However, the magnitude of the mass change was too small to affect the other muscles significantly. Fifth, age differences were significant in comparing the older and younger groups. Older adults took longer and exerted more effort to complete the task than younger adults. Static exercise in older adults may be more taxing on muscle activity in old age than powered exercise, whereas young adults are more dominant in powered exercise.

### 4.2 Other factors

From Table 9, the horizontal distance of the body from the hanger has a very significant effect on the angle of the body joints studied, and somehow this horizontal distance can be converted with the depth design parameters of the hanger. The variable of the need for external augmentation was addressed during the experiment by adding pads to supplement the participants’ height, which affected the body’s lower extremities, including the ankle, knee, and hip joints. In contrast, the upper extremities required attention to wrist, shoulder, and torso. The effect of gender factors is complicated, and this is more of the effect of raw physiological differences between men and women, such as height, arm length, and other physical data (Slopecki et al., 2020; Firouzabadi et al., 2021). The main effect of the object mass factor did not reach significance.

**TABLE 9:** Between-subjects effect of different variable factors on joint angles.

Variable	L.Wrist	L.Elbow	L.Shoulder	R.Wrist	R.Elbow	R.Shoulder	Neck	Trunk
Distance	9.83**	12.32**	22.69**	30.88**	8.57**	34.68**	2.86*	18.52**
Posture	0.952	14.54**	17.7**	1.67	1.26	2.33	0.749	0.084
Mat	12.4**	0.043	9.77**	32.56**	0.157	58.39**	1.519	44.06**
Gender	3.616	4.76*	6.38*	24.23**	8.1**	1.775	0.802	37.99**
Mass	1.257	0.283	0.053	0.299	0.46	0.096	0.072	0.012
**Variable**	**L.Foot**	**L.Ankle**	**L.Knee**	**.L.Hip**	**R.Foot**	**R.Ankle**	**R.Knee**	**R.Hip**
Distance	5.53**	15.82**	17.24**	13.26**	38.91**	31.34**	10.09**	6.39**
Posture	3.86**	4.61**	2.93*	10.65**	5.63**	1.623	2.65*	9.57**
Mat	1.668	54.84**	34.54**	45.88**	1.055	30.46**	25.65**	38.74**
Gender	1.528	2.751	2.398	12.75**	1.29	0.225	5.3*	14.17**
Mass	1.203	0.442	0.353	0.379	1.732	0.155	1.025	0.387

“*”: indicates significant correlation at the 0.05 level (two-tailed).

Regarding the two factors of horizontal distance between the body and the lift and the need for external augmentation, external augmentation is needed when the height is less than 150 cm, but changing the height of the lift is also an option, and proper adjustment of the height of the lift can avoid physical injury. Muscle activity levels are also generally higher when loads are higher (Martinez et al., 2022). Table 4 shows the relationship between height range and horizontal distance. The optimal holding distance is 10 cm when the height range is between 154 and 157 cm, 20 cm when the height range is between 158 and 167 cm, 30 cm when the height range is between 168 and 177 cm, and 40 cm when the height range is 178 cm and above. The results of the study showed that at the same height of the hanging cabinet, increasing the horizontal distance from the hanging cabinet increases the range of motion of the joints. The appropriate distance from the hanging cabinet can increase the range of motion of the joints to reduce the force when picking up heavy objects, and it is not suitable to be too big or too small.

Overall, age greatly affected access to high objects, followed by height. Different heights affected the participants’ comfort status when using the hanging cabinets. First, in terms of the height of the hanging cabinet, the difference in human height translates into the height of the hanging cabinet, which needs to be less than 180 cm if the human height is less than 150 cm, while the optimal height range is between 120 and 150 cm. Second, in terms of the depth of the hanging cabinet, participants of different heights chose different comfort distances, and this distance was translated into the depth of the hanging cabinet, with different height ranges corresponding to the depth of the hanging cabinet (Hu and Chen, 2021). The greater the height, the greater the depth of the wall cabinet that can be used, with an optimal depth range of 30–35 cm.

### 4.3 Comfort evaluation

**FIGURE 6:**
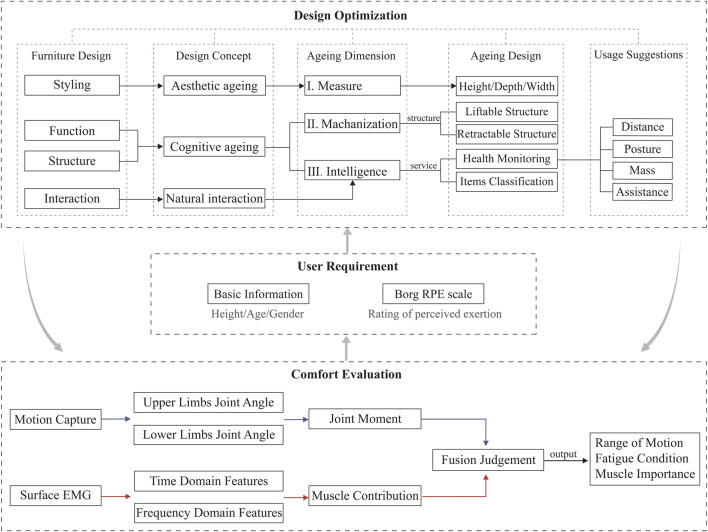
Comfort evaluation model of hanging cabinet.

We focus on human comfort from a kinetic point of view and a kinematic point of view, exploring human comfort in conjunction with surface electromyography (sEMG) and joint motion fusion (e.g., Figure 6). The experimental data were normalized with normalization values ranging from 1 to 10. A polynomial fitting function was used to fit the normalized data polynomially. A postural discomfort model was established with the change in joint movement angle and the postural discomfort index as the dependent variable. 1) Establishment of factor set. In fetching objects from a high place, the human upper limb posture discomfort is affected by the discomfort of five joints, which are selected as wrist joint, elbow joint, shoulder joint, neck joint, and lumbar spine joint. The factor set is established as *U* = {*u*_1_, *u*_2_, *u*_3_, *u*_4_, *u*_5_}, where *u*_1_ is the wrist joint discomfort, *u*_2_ is the elbow joint discomfort, *u*_3_ is the shoulder joint discomfort, *u*_4_ is the neck joint discomfort, and *u*_5_ is the lumbar spine joint discomfort. 2) Establishment of weight sets. Kee and Karwowski (2001) utilized the free modulus method to classify the degree of influence of human joints on postural discomfort. The weight coefficients of each joint were determined using the AHP method according to different grades, with higher weight coefficients indicating a greater degree of influence on comfort. The weight coefficients of each joint part are the wrist joint (0.076), elbow joint (0.231), shoulder joint (0.231), neck (0.154), and torso (0.308). 3) Establishment of evaluation sets. An evaluation set is a collection of possible results from the evaluation object. The possible evaluation results of upper extremity postural discomfort are categorized into “very comfortable,” “more comfortable,” “general,” “less comfortable, “and “very uncomfortable.” The evaluation set is *V* = {*v*_1_, *v*_2_, *v*_3_, *v*_4_, *v*_5_}. Its value ranges between [0, 10], and the subjective evaluation method was used to grade the postural discomfort composite index into five levels, where [0, 2] is very comfortable, (2, 4] is more comfortable, (4, 6] is average, (6, 8] is less comfortable, and (8, 10] is very uncomfortable. Based on the magnitude of the composite index of joint movement discomfort, its affiliation to each element in the alternative set *V* is derived. 4) Fuzzy comprehensive evaluation. If the affiliation degree of the *i*th element in the factor set *U* to the first element in the evaluation set *V* is *r*_*i*1_, the result of the single-factor evaluation of the *i*th element is expressed as a fuzzy set as *R*_*i*_ = [*r*_*i*1_, *r*_*i*2_, …, *r*_*in*_], and the matrix *R*_m*n_ is formed with the rows of the m single-factor evaluation sets *R*_1_, *R*_2_, …, *R*_*m*_. The fuzzy evaluation formula for upper extremity postural discomfort is obtained: *C* = *A*°*R*_*i*_. *A* is the weighting coefficient of each joint, *C* is the upper extremity postural discomfort evaluation result vector, and “°” is the weighted average operator *M*(⋅, +).

According to the above model, we can obtain the comfort indexes of the left and right hemispheres. The resulting vector of upper limb posture discomfort evaluation is obtained by the joint movement angle while fetching objects from a high place. For the example of Posture 03, its upper extremity discomforts are “very comfortable, more comfortable, average, less comfortable, very uncomfortable,” with the membership degree of “.0540, .2884,0, .3592, 0”, respectively. According to the principle of maximum affiliation, the posture is uncomfortable. Based on the widely used RULA evaluation method, the result of this posture was three points with a grade of 2.

### 4.4 Hanging cabinet evaluation and optimization

From a kinetic point of view, the joint angle reflects the range of joint motion of the human body. The smaller the range of variation of the joints, the lower the intensity of limb activity, i.e., the limb is relatively comfortable. The joint moments can visualise the loads on the joints, which can also evaluate the joints’ comfort; when picking up objects from a high place, the maximum and minimum values at the joints alternate. The attenuation of peaks is an essential step in optimal furniture design or posture optimisation. From a kinematic point of view, muscle activity is positively correlated with EMG signal density. The iEMG in time domain analysis reflects the strength of muscle activity over a while, and the greater the amplitude, the greater the muscle fatigue. The muscle contribution index (calculated from the iEMG) can determine the magnitude of the degree of muscle force generation and classify the muscle contribution level. Based on sEMG and motion capture system, the muscle and joint comfort evaluation process integrate multiple biological and physiological signals (Chao et al., 2023), effectively improves movement and behaviour analysis accuracy, and evaluates fatigue and comfort during furniture use.

The study results showed that alternating between the right and left hand for retrieving objects from high places (one hand interacting with the cabinet door and the other hand interacting with the heavy object) was significantly advantageous and more in line with human comfort requirements. Biceps muscle activity was maximal when interacting with the cabinet door (maximal during the cabinet door opening phase); trapezius muscle activity was maximal when interacting with the heavy object. Second, in older adults, static movements may be more demanding on muscle activity than powered movements. In addition, the human height translates into the height of the hanging cabinet, which must be less than 180 cm if the human height is less than 150 cm. In contrast, the optimal height range is between 120 and 150 cm. In terms of the depth of the cabinet, participants of different heights chose different comfort distances, and this distance is translated into the depth of the cabinet, with different height increments corresponding to the depth of the cabinet. The greater the height, the greater the depth of the wall cabinet that can be used, with an optimal depth range of 30–35 cm.

Finally, the optimal design of hanging cabinets must fully account for the user’s physiological information and subjective perception. Basic information such as height and age are the key to defining the dimensions of a hanging cabinet. At the same time, subjective fatigue perception creates many limitations for users when using a hanging cabinet, such as the requirements for holding posture, the suggestion of appropriate mass, and the need for external assistance (Fang and Shen, 2022). Therefore, in the design optimisation of the hanging cabinet furniture, the shape was defined from aesthetic aging (Xiong et al., 2020), the basic dimension was reflected in size, and the optimal recommendations of the hanging cabinet regarding height and depth were obtained through experiments. From perceptual aging defining function and structure (Zhu and Hou, 2021), the basic dimension is reflected in mechanisation and intelligence (Wang et al., 2023), adapting to the different heights of members of the family to choose lifting and retractable hanging cabinets, adapting to the age and health of different members, and remembering the mass of objects is also essential. Furthermore, this advice on using hanging cabinets reflects the importance of a smart home.

### 4.5 Limitations and further work

The limitations of this study include two categories: experimental analysis and data analysis. From the experimental point of view, the first point is that the height of the hanging cabinet was set to a fixed value of 2 m for the experiment, which rounded off the study of this variable, and changes in different heights may have many effects on the results (Blache et al., 2015a). The second point is that the range of mass thresholds for picking up objects from a high place is too small, and the 0.5 kg increment may not fully reflect the difference and may only affect some sensitive muscle positions. As for the third point, the accuracy of data recording could not be tested. Finally, the fixed experimental posture and distance will affect the data recording of different individuals, which is more idealised and fixed than hanging cabinets in daily life. From the data analysis: The first point, we analyse the mechanical data of the participants by using the average body size and weight of the older participants in this experiment, which may bring bias due to the differences of different individuals, but the effect on the overall results may be small. The range of human heights in this experiment was evenly distributed, and the design principles for hanging cabinets were summarised after considering many possibilities that apply to the design of hanging cabinets for older adults in general. The second point is that the effects of other factors on the experimental data were out of consideration in the experiment, such as the EMG changes caused by the participants’ cognitive distraction while performing the task. However, this effect is recognised as a benign one (Joseph et al., 2014). It is also considered that different completion speeds may also affect the data (Yoon et al., 2012). Finally, the integration of subjective analysis is lacking, with experiments assessing subjective fatigue in participants using the Borg Subjective Fatigue Perception Assessment Scale (Arney et al., 2019; Antwi-Afari et al., 2021), which can be mapped to EMG and motion capture data results. Future research should be expanded to examine the effects of other variables and attempt to study the behaviour of older adults with other types of furniture to provide a complete assessment and validation of ergonomics and furniture design after translating theory into practice.

For further work, it is necessary to consider more groups of older adults with different limiting conditions, such as physical disabilities associated with age increases that appear quite different when using furniture. Older adults with MSD have had chronic pain problems over the years, including arm and low back pain, which can interfere with research on the spine or other joints. In addition, there is a need for future research to include subjective fatigue perception in the comfort evaluation and to study the behaviour of other furniture uses further, applying it to practice to provide a complete assessment and validation of ergonomics and furniture design.

## 5 Conclusion

This study aimed to understand whether different loads and retrieving postures during high-level picking affect muscle activity and joint ROM to provide optimised feedback for the design of hanging cabinet furniture. The study results showed that: 1) the activity of BB was greater than that of UT and BR on the side of the body interacting with the cabinet door during overhead retrieving. The activity of UT was greater than that of BB and BR on the side of the body interacting with the object. 2) the activity of UT decreased when the shoulder joint movement was greater than 90°. However, the activity of BB increased with the angle, while an increase in the participants’ mass induced a maximal load on the shoulder joint. 3) Complete two-handed and one-handed task performance resulted in greater muscle activity and moments and greater changes in joint angle than alternating between the right and left hand. 4) Age had the greatest effect on reaching from a height, followed by body size. Mass changes in the experiment were significantly different only at the left elbow joint and the left brachioradialis.

From these results, it is clear that: 1) of the different postures for retrieving objects from a height, alternating between the right and left hand for retrieving objects from height is more desirable. Maximum effort was observed in the phase of picking up heavy objects and in the phase of opening the cabinet door during the movement. 2) Gender and object mass differences had little effect on the low-load task, whereas height differences affected the participants’ comfort level when using the hanging cabinet. 3) Older adults took longer and exerted more effort to complete the task than younger adults, and static exercise in older adults may be more demanding on muscle activity in old age than powered exercise.

The major contributions of the study are:

1) To provide a complete muscle and joint comfort evaluation process based on sEMG and motion capture system, which effectively improves the accuracy of motion and behaviour analysis during hanging cabinet furniture use.
2) To contribute to the design optimisation and installation of the hanging cabinet. In terms of the installation height of the hanging cabinet, if the human height is less than 1,500 mm, the height of the hanging cabinet bottom plate must be less than 1,800 mm to complete the normal retrieving action, and the optimal height range of the bottom plate is between 1,200 and 1,500 mm. From the design of the cabinet itself, participants of different heights chose different comfortable distances, translating into the cabinet’s depth. The higher the participant’s height, the greater the depth of the hanging cabinet available. The best depth range is between 300 and 350 mm.
3) Defining the function and structure of hanging cabinet furniture from perceived aging, the underlying dimensions are reflected in mechanisation and intelligence, adapting to the different heights of members of the family choosing lifting and retractable hanging cabinets, adapting to the age and health of different members of the family.

## Data Availability

The raw data supporting the conclusion of this article will be made available by the authors, without undue reservation.

## Abbreviations

AD, Anterior Deltoid; BB, Biceps Brachii; BR, Brachial Radius; CORB, Coracobrachialis; ECR, Extensor Carpi Radialis; ECU, Extensor Carpi Ulnaris; ED, Extensor Digitorum; EDC, Extensor Digitorum Communis; FCR, Flexor Carpi Radialis; FDS, Flexor Digitorum Superficialis; INF, Infraspinatus; ITR, Inferior Trapezius; LD, Latissimus Dorsi; LVS, Levator Scapulae; MD, Middle Deltoid; MES, Middle Erector Spinae; MTR, Middle Trapezius; PD, Posterior Deltoid; PM, Pectoralis Major; RM, Rhomboid Major; SA, Serratus Anterior; STR, Superior Trapezius; TB, Triceps Brachii; TR, Trapezius; UT, Upper Trapezius.

## References

[B1] Amarantini D. Martin L. (2004). A method to combine numerical optimization and emg data for the estimation of joint moments under dynamic conditions. J. Biomechanics 37, 1393–1404. 10.1016/j.jbiomech.2003.12.020 15275847

[B2] Antony N. T. Keir P. J. (2010). Effects of posture, movement and hand load on shoulder muscle activity. J. Electromyogr. Kinesiol. 20, 191–198. 10.1016/j.jelekin.2009.04.010 19473855

[B3] Antwi-Afari M. F. Li H. Anwer S. Li D. W. Yu Y. Mi H. Y. (2021). Assessment of a passive exoskeleton system on spinal biomechanics and subjective responses during manual repetitive handling tasks among construction workers. Saf. Sci. 142, 105382. 10.1016/j.ssci.2021.105382

[B4] Arjmand N. Shirazi-Adl A. (2005). Biomechanics of changes in lumbar posture in static lifting. Spine 30, 2637–2648. 10.1097/01.brs.0000187907.02910.4f 16319750

[B5] Arjunan S. P. Kumar D. K. (2013). Age-associated changes in muscle activity during isometric contraction. Muscle & Nerve 47, 545–549. 10.1002/mus.23619 23203513

[B6] Arney B. E. Glover R. Fusco A. Cortis C. de Koning J. J. van Erp T. (2019). Comparison of rpe (rating of perceived exertion) scales for session rpe. Int. J. Sports Physiology Perform. 14, 994–996. 10.1123/ijspp.2018-0637 30569764

[B7] Au A. K. Keir P. J. (2007). Interfering effects of multitasking on muscle activity in the upper extremity. J. Electromyogr. Kinesiol. 17, 578–586. 10.1016/j.jelekin.2006.06.005 16904910

[B8] Avin K. G. Law L. A. F. (2011). Age-related differences in muscle fatigue vary by contraction type: a meta-analysis. Phys. Ther. 91, 1153–1165. 10.2522/ptj.20100333 21616932 PMC3145894

[B9] Bazazan A. Dianat I. Feizollahi N. Mombeini Z. Shirazi A. M. Castellucci H. I. (2019). Effect of a posture correction–based intervention on musculoskeletal symptoms and fatigue among control room operators. Appl. Ergon. 76, 12–19. 10.1016/j.apergo.2018.11.008 30642516

[B10] Beaucage-Gauvreau E. Brandon S. C. E. Robertson W. S. P. Fraser R. Freeman B. J. C. Graham R. B. (2021). Lumbar spine loads are reduced for activities of daily living when using a braced arm-to-thigh technique. Eur. Spine J. 30, 1035–1042. 10.1007/s00586-020-06631-0 33156439

[B11] Bernard B. P. Putz-Anderson V. (1997). Musculoskeletal disorders and workplace factors; a critical review of epidemiologic evidence for work-related musculoskeletal disorders of the neck, upper extremity, and low back. Cincinnati, US: National Institute for Occupational Safety and Health.

[B12] Bieleman H. J. Rijken N. H. M. Reneman M. F. Oosterveld F. G. J. Soer R. (2021). Changes in kinematics and work physiology during progressive lifting in healthy adults. Appl. Ergon. 94, 103396. 10.1016/j.apergo.2021.103396 33667899

[B13] Bilodeau M. Henderson T. K. Nolta B. E. Pursley P. J. Sandfort G. L. (2001). Effect of aging on fatigue characteristics of elbow flexor muscles during sustained submaximal contraction. J. Appl. Physiology 91, 2654–2664. 10.1152/jappl.2001.91.6.2654 11717231

[B14] Blache Y. Dal Maso F. Desmoulins L. Plamondon A. Begon M. (2015a). Superficial shoulder muscle co-activations during lifting tasks: influence of lifting height, weight and phase. J. Electromyogr. Kinesiol. 25, 355–362. 10.1016/j.jelekin.2014.11.004 25483204

[B15] Blache Y. Desmoulins L. Allard P. Plamondon A. Begon M. (2015b). Effects of height and load weight on shoulder muscle work during overhead lifting task. Ergonomics 58, 748–761. 10.1080/00140139.2014.980336 25403553

[B16] Bonnet C. T. Barela J. A. (2021). Health issues due to the global prevalence of sedentariness and recommendations towards achieving a healthier behaviour. Healthcare 9, 995. 10.3390/healthcare9080995 34442132 PMC8394200

[B17] Bouffard J. Martinez R. Plamondon A. Cote J. N. Begon M. (2019). Sex differences in glenohumeral muscle activation and coactivation during a box lifting task. Ergonomics 62, 1327–1338. 10.1080/00140139.2019.1640396 31282824

[B18] Brookham R. L. Wong J. M. Dickerson C. R. (2010). Upper limb posture and submaximal hand tasks influence shoulder muscle activity. Int. J. Industrial Ergonomics 40, 337–344. 10.1016/j.ergon.2009.11.006

[B19] Bryanton M. A. Bilodeau M. (2019). The influence of knee extensor fatigue on lower extremity muscle activity during chair rise in young and older adults. Eur. J. Appl. Physiology 119, 61–71. 10.1007/s00421-018-3999-4 30317389

[B20] Castelein B. Cools A. Parlevliet T. Cagnie B. (2017). The influence of induced shoulder muscle pain on rotator cuff and scapulothoracic muscle activity during elevation of the arm. J. Shoulder Elb. Surg. 26, 497–505. 10.1016/j.jse.2016.09.005 27751718

[B21] Cattarello P. Vinelli S. D’Emanuele S. Gazzoni M. Merletti R. (2018). Comparison of chairs based on hdsemg of back muscles, biomechanical and comfort indices, for violin and viola players: a short-term study. J. Electromyogr. Kinesiol. 42, 92–103. 10.1016/j.jelekin.2018.06.013 30015135

[B22] Chang S. J. Kim H. J. Juon H. S. Park H. Choi S. W. Lee K. E. (2022). A comparison of the influencing factors of chronic pain and quality of life between older koreans and Korean-americans with chronic pain: a correlational study. Qual. Life Res. 31, 1179–1189. 10.1007/s11136-021-02983-2 34462905 PMC8960560

[B23] Chao Y. Liu T. Shen L. M. (2023). Method of recognizing sleep postures based on air pressure sensor and convolutional neural network: for an air spring mattress. Eng. Appl. Artif. Intell. 121, 106009. 10.1016/j.engappai.2023.106009

[B24] Christensen S. W. M. Palsson T. S. Krebs H. J. Graven-Nielsen T. Hirata R. P. (2023). Prolonged slumped sitting causes neck pain and increased axioscapular muscle activity during a computer task in healthy participants-a randomized crossover study. Appl. Ergon. 110, 104020. 10.1016/j.apergo.2023.104020 36958253

[B25] De Carvalho D. E. Callaghan J. P. (2022). Effect of office chair design features on lumbar spine posture, muscle activity and perceived pain during prolonged sitting. Ergonomics 66, 1465–1476. 10.1080/00140139.2022.2152113 36437777

[B26] DePalma M. J. Ketchum J. M. Saullo T. R. (2012). Multivariable analyses of the relationships between age, gender, and body mass index and the source of chronic low back pain. Pain Med. 13, 498–506. 10.1111/j.1526-4637.2012.01339.x 22390231

[B27] Deschenes M. R. (2004). Effects of aging on muscle fibre type and size. Sports Med. 34, 809–824. 10.2165/00007256-200434120-00002 15462613

[B28] Docking R. E. Fleming J. Brayne C. Zhao J. Macfarlane G. J. Jones G. T. (2011). Epidemiology of back pain in older adults: prevalence and risk factors for back pain onset. Rheumatology 50, 1645–1653. 10.1093/rheumatology/ker175 21606130

[B29] Elsayed W. Farrag A. El-Sayyad M. Marras W. (2015). Changes in muscular activity and lumbosacral kinematics in response to handling objects of unknown mass magnitude. Hum. Mov. Sci. 40, 315–325. 10.1016/j.humov.2015.01.008 25662505

[B30] Erdemir A. McLean S. Herzog W. van den Bogert A. J. (2007). Model-based estimation of muscle forces exerted during movements. Clin. Biomech. 22, 131–154. 10.1016/j.clinbiomech.2006.09.005 17070969

[B31] Etherton J. Lawson M. Graham R. (2014). Individual and gender differences in subjective and objective indices of pain: gender, fear of pain, pain catastrophizing and cardiovascular reactivity. Appl. Psychophysiol. Biofeedback 39, 89–97. 10.1007/s10484-014-9245-x 24696322

[B32] Fang J. J. Shen L. M. (2022). Analysis of sagittal spinal alignment at the adolescent age: for furniture design. Ergonomics 17doi, 1477–1493. 10.1080/00140139.2022.2152491 36437772

[B33] Fang N. L. Zhang C. Lv J. (2021). Effects of vertical lifting distance on upper-body muscle fatigue. Int. J. Environ. Res. Public Health 18, 5468. 10.3390/ijerph18105468 34065333 PMC8160884

[B34] Firouzabadi A. Arjmand N. Pan F. M. Zander T. Schmidt H. (2021). Sex-dependent estimation of spinal loads during static manual material handling activities-combined *in vivo* and *in silico* analyses. Front. Bioeng. Biotechnol. 9, 750862. 10.3389/fbioe.2021.750862 34796167 PMC8592996

[B35] Freburger J. K. Holmes G. M. Agans R. P. Jackman A. M. Darter J. D. Wallace A. S. (2009). The rising prevalence of chronic low back pain. Archives Intern. Med. 169, 251–258. 10.1001/archinternmed.2008.543 PMC4339077 19204216

[B36] Goncalves M. A. Arezes P. M. (2012). Postural assessment of school children: an input for the design of furniture. Work-a J. Prev. Assess. Rehabilitation 41, 876–880. 10.3233/wor-2012-0257-876 22316832

[B37] Goyal A. K. Mohanty S. K. (2022). Association of pain and quality of life among middle-aged and older adults of India. Bmc Geriatr. 22, 939. 10.1186/s12877-022-03480-y 36474187 PMC9724285

[B38] Granata K. P. Gottipati P. (2008). Fatigue influences the dynamic stability of the torso. Ergonomics 51, 1258–1271. 10.1080/00140130802030722 18608477

[B39] Granata K. P. Wilson S. E. (2001). Trunk posture and spinal stability. Clin. Biomech. 16, 650–659. 10.1016/s0268-0033(01)00064-x 11535346

[B40] Granata K. R. Slota G. R. Wilson S. E. (2004). Influence of fatigue in neuromuscular control of spinal stability. Hum. Factors 46, 81–91. 10.1518/hfes.46.1.81.30391 15151156 PMC1633714

[B41] Hoogendoorn W. E. Bongers P. M. De Vet H. C. Douwes M. Koes B. W. Miedema M. C. (2000). Flexion and rotation of the trunk and lifting at work are risk factors for low back pain: results of a prospective cohort study. Spine 25, 3087–3092. 10.1097/00007632-200012010-00018 11145822

[B42] Hortobagyi T. Mizelle C. Beam S. DeVita P. (2003). Old adults perform activities of daily living near their maximal capabilities. Journals Gerontology Ser. a-Biological Sci. Med. Sci. 58, 453–460. 10.1093/gerona/58.5.M453 12730256

[B43] Hoy D. Bain C. Williams G. March L. Brooks P. Blyth F. (2012). A systematic review of the global prevalence of low back pain. Arthritis Rheumatism 64, 2028–2037. 10.1002/art.34347 22231424

[B44] Hoy D. Brooks P. Blyth F. Buchbinder R. (2010). The epidemiology of low back pain. Best Pract. Res. Clin. Rheumatology 24, 769–781. 10.1016/j.berh.2010.10.002 21665125

[B45] Hu W. G. Chen B. R. (2021). A methodology for optimizing tenon geometry dimensions of mortise-and-tenon joint wood products. Forests 12, 478. 10.3390/f12040478

[B46] Johannes C. B. Le T. K. Zhou X. L. Johnston J. A. Dworkin R. H. (2010). The prevalence of chronic pain in United States adults results of an internet-based survey. J. Pain 11, 1230–1239. 10.1016/j.jpain.2010.07.002 20797916

[B47] Joseph C. Beach T. A. C. Callaghan J. P. Dickerson C. R. (2014). The influence of precision requirements and cognitive challenges on upper extremity joint reaction forces, moments and muscle force estimates during prolonged repetitive lifting. Ergonomics 57, 236–246. 10.1080/00140139.2013.869359 24437984

[B48] Kak D. W. Anita A. R. Nizlan N. M. Normala I. Jalil N. A. A. Wong S. V. (2019). Comparison of neck muscle electromyography activity in response to external force between static and dynamic loading. J. Mech. Med. Biol. 19, 1850034. 10.1142/s0219519418500343

[B49] Kazemi Z. Mazloumi A. Arjmand N. Keihani A. Karimi Z. Ghasemi M. S. (2022). A comprehensive evaluation of spine kinematics, kinetics, and trunk muscle activities during fatigue-induced repetitive lifting. Hum. Factors 64, 997–1012. 10.1177/0018720820983621 33497290

[B50] Kee D. Karwowski W. (2001). Luba: an assessment technique for postural loading on the upper body based on joint motion discomfort and maximum holding time. Appl. Ergon. 32, 357–366. 10.1016/S0003-6870(01)00006-0 11461037

[B51] Keir P. J. Brown M. M. (2012). Force, frequency and gripping alter upper extremity muscle activity during a cyclic push task. Ergonomics 55, 813–824. 10.1080/00140139.2012.668947 22506613

[B52] Kern D. S. Semmler J. G. Enoka R. M. (2001). Long-term activity in upper- and lower-limb muscles of humans. J. Appl. Physiology 91, 2224–2232. 10.1152/jappl.2001.91.5.2224 11641365

[B53] Kim J. Y. Chung M. K. Park J. S. (2003). Measurement of physical work capacity during arm and shoulder lifting at various shoulder flexion and ad/abduction angles. Hum. Factors Ergonomics Manuf. 13, 153–163. 10.1002/hfm.10034

[B54] Larsson C. Hansson E. E. Sundquist K. Jakobsson U. (2017). Chronic pain in older adults: prevalence, incidence, and risk factors. Scand. J. Rheumatology 46, 317–325. 10.1080/03009742.2016.1218543 27885914

[B55] Lewanska M. Grzegorzewski A. Walusiak-Skorupa J. (2016). Bilateral hypermobility of ulnar nerves at the elbow joint with unilateral left ulnar neuropathy in a computer user: a case study. Int. J. Occup. Med. Environ. Health 29, 517–522. 10.13075/ijomeh.1896.00398 26988889

[B56] Madinei S. Ning X. P. (2018). Effects of the weight configuration of hand load on trunk musculature during static weight holding. Ergonomics 61, 831–838. 10.1080/00140139.2017.1387675 28965479 PMC5929471

[B57] Marras W. S. Parakkat J. Chany A. M. Yang G. Burr D. Lavender S. A. (2006). Spine loading as a function of lift frequency, exposure duration, and work experience. Clin. Biomech. 21, 345–352. 10.1016/j.clinbiomech.2005.10.004 16310299

[B58] Martinez K. B. Nazarahari M. Rouhani H. (2022). K-score: a novel scoring system to quantify fatigue-related ergonomic risk based on joint angle measurements via wearable inertial measurement units. Appl. Ergon. 102, 7. 10.1016/j.apergo.2022.103757 35378482

[B59] Martinez R. Assila N. Goubault E. Begon M. (2020). Sex differences in upper limb musculoskeletal biomechanics during a lifting task. Appl. Ergon. 86, 103106. 10.1016/j.apergo.2020.103106 32342895

[B60] Millard M. Emonds A. L. Harant M. Mombaur K. (2019). A reduced muscle model and planar musculoskeletal model fit for the simulation of whole-body movements. J. Biomechanics 89, 11–20. 10.1016/j.jbiomech.2019.04.004 31000347

[B61] Nielsen P. K. Andersen L. Jorgensen K. (1998). The muscular load on the lower back and shoulders due to lifting at different lifting heights and frequencies. Appl. Ergon. 29, 445–450. 10.1016/s0003-6870(98)00005-2 9796790

[B62] Norin L. Slaug B. Haak M. Iwarsson S. (2021). Housing adaptations and housing accessibility problems among older adults with long-standing spinal cord injury. Br. J. Occup. Ther. 84, 765–774. 10.1177/0308022620979516

[B63] Okajima S. Costa-Garcia A. Ueda S. Yang N. J. Shimoda S. (2023). Forearm muscle activity estimation based on anatomical structure of muscles. Anatomical Record-Advances Integr. Anat. Evol. Biol. 306, 741–763. 10.1002/ar.24910 35385221

[B64] Rasmussen J. Torholm S. de Zee M. (2009). Computational analysis of the influence of seat pan inclination and friction on muscle activity and spinal joint forces. Int. J. Industrial Ergonomics 39, 52–57. 10.1016/j.ergon.2008.07.008

[B65] Richter-Kluge J. Wellhausen C. Frese U. (2019). “Esko6d-a binocular and rgb-d dataset of stored kitchen objects with 6d posesesko6d-a,” in IEEE/RSJ International Conference on Intelligent Robots and Systems (IROS), Ieee, 893. –899. 10.1109/IROS40897.2019.8967937

[B66] Rohlmann A. Zander T. Graichen F. Bergmann G. (2013). Lifting up and laying down a weight causes high spinal loads. J. Biomechanics 46, 511–514. 10.1016/j.jbiomech.2012.10.022 23141957

[B67] Roldan-Jimenez C. Bennett P. Cuesta-Vargas A. I. (2015). Muscular activity and fatigue in lower-limb and trunk muscles during different sit-to-stand tests. Plos One 10, e0141675. 10.1371/journal.pone.0141675 26506612 PMC4624782

[B68] Roman-Liu D. Tokarski T. (2005). Upper limb strength in relation to upper limb posture. Int. J. Industrial Ergonomics 35, 19–31. 10.1016/j.ergon.2004.07.002

[B69] Saito H. Yokoyama H. Sasaki A. Kato T. Nakazawa K. (2022). Evidence for basic units of upper limb muscle synergies underlying a variety of complex human manipulations. J. Neurophysiology 127, 958–968. 10.1152/jn.00499.2021 35235466

[B70] Sawaguchi R. Tanaka H. (2019). Visualization of the muscle tension in stand-up and sit-down motion using mocap and emg. Adv. Phys. Ergonomics Hum. Factors 789, 196–207. 10.1007/978-3-319-94484-5_21

[B71] Shi X. A. Zhang F. (2023). Analysis of the hanging actions and operating heights of storage furniture suitable for the elderly. Sensors 23, 3850. 10.3390/s23083850 37112191 PMC10145950

[B72] Simundic V. Dzijan M. Pejic P. Cupec R. (2023). Introduction to door opening type classification based on human demonstration. Sensors 23, 3093. 10.3390/s23063093 36991804 PMC10051927

[B73] Skals S. Blafoss R. Andersen M. S. de Zee M. Andersen L. L. (2021). Manual material handling in the supermarket sector. part 1: joint angles and muscle activity of trapezius descendens and erector spinae longissimus. Appl. Ergon. 92, 103340. 10.1016/j.apergo.2020.103340 33340719

[B74] Slopecki M. Messing K. Cote J. N. (2020). Is sex a proxy for mechanical variables during an upper limb repetitive movement task? an investigation of the effects of sex and of anthropometric load on muscle fatigue. Biol. Sex Differ. 11, 60. 10.1186/s13293-020-00336-1 33126920 PMC7596960

[B75] Tikkanen O. Sipila S. Kuula A. S. Pesola A. Haakana P. Finni T. (2016). Muscle activity during daily life in the older people. Aging Clin. Exp. Res. 28, 713–720. 10.1007/s40520-015-0482-5 26526027

[B76] Treede R. D. Rief W. Barke A. Aziz Q. Bennett M. I. Benoliel R. (2015). A classification of chronic pain for icd-11. Pain 156, 1003–1007. 10.1097/j.pain.0000000000000160 25844555 PMC4450869

[B77] Tsai L. C. Chen S. C. Chen Y. C. Lee L. Y. (2022). The impact of physical pain and depression on sleep quality in older adults with chronic disease. J. Clin. Nurs. 31, 1389–1396. 10.1111/jocn.16000 34498323

[B78] Tsang A. Von Korff M. Lee S. Alonso J. Karam E. Angermeyer M. C. (2008). Common chronic pain conditions in developed and developing countries: gender and age differences and comorbidity with depression-anxiety disorders. J. Pain 9, 883–891. 10.1016/j.jpain.2008.05.005 18602869

[B79] Umehara J. Yagi M. Hirono T. Ueda Y. Ichihashi N. (2021). Quantification of muscle coordination underlying basic shoulder movements using muscle synergy extraction. J. Biomechanics 120, 110358. 10.1016/j.jbiomech.2021.110358 33743396

[B80] Vandervoort A. A. (2002). Aging of the human neuromuscular system. Muscle & Nerve 25, 17–25. 10.1002/mus.1215 11754180

[B81] Wang J. Wu X. Y. Wang Y. J. Zhao W. Y. Zhao Y. Zhou M. (2023). Green, sustainable architectural bamboo with high light transmission and excellent electromagnetic shielding as a candidate for energy-saving buildings. Nano-Micro Lett. 15, 11. 10.1007/s40820-022-00982-7 PMC9741695 36495422

[B82] Wettstein M. Tesarz J. (2023). Increasing pain prevalence and intensity among middle-aged and older adults: evidence from the German ageing survey. J. Psychosomatic Res. 168, 111233. 10.1016/j.jpsychores.2023.111233 36958227

[B83] Xiong X. Q. Ma Q. R. Yuan Y. Y. Wu Z. H. Zhang M. (2020). Current situation and key manufacturing considerations of green furniture in China: a review. J. Clean. Prod. 267, 15. 10.1016/j.jclepro.2020.121957

[B84] Yoon J. Shiekhzadeh A. Nordin M. (2012). The effect of load weight vs. pace on muscle recruitment during lifting. Appl. Ergon. 43, 1044–1050. 10.1016/j.apergo.2012.03.004 22475433

[B85] Yu N. Wu P. W. (2019). Effect of physical fatigue on cognitive ability of workers in furniture cnc operation. Man-Machine-Environment Syst. Eng. Mmese 527, 49–55. 10.1007/978-981-13-2481-9_7

[B86] Zhu J. G. Hou H. P. (2021). Research on user experience evaluation of mobile applications in government services. Ieee Access 9, 52634–52641. 10.1109/access.2021.3070365

[B87] Zis P. Daskalaki A. Bountouni I. Sykioti P. Varrassi G. Paladini A. (2017). Depression and chronic pain in the elderly: links and management challenges. Clin. Interventions Aging 12, 709–720. 10.2147/cia.s113576 PMC5407450 28461745

